# Empirical modeling and prediction of neuronal dynamics

**DOI:** 10.1007/s00422-024-00986-z

**Published:** 2024-04-10

**Authors:** Pau Fisco-Compte, David Aquilué-Llorens, Nestor Roqueiro, Enric Fossas, Antoni Guillamon

**Affiliations:** 1 Departament d’Enginyeria Elèctrica, CITCEA-UPC, Universitat Politècnica de Catalunya - Barcelona TECH, Av. Diagonal, 647, (Edifici ETSEIB), Barcelona, Catalonia, 08028 Spain; 2 Neuroscience BU, Starlab Barcelona S.L., Av Tibidabo 47 bis, Barcelona, Catalonia, 08035 Spain; 3 Depto. de Automação e Sistemas, Federal University of Santa Catarina, Bairro Trindade, Caixa Postal 476, Florianopolis, Santa Catarina 88040-900 Brazil; 4 Institut d’Organització i Control, Universitat Politècnica de Catalunya - Barcelona TECH, Av. Diagonal, 647, planta 11 (Edifici ETSEIB), Barcelona, Catalonia, 08028 Spain; 5 Departament de Matemàtiques (EPSEB) and Institut de Matemàtiques de la UPC (IMTech), Universitat Politècnica de Catalunya - Barcelona TECH, Av. Dr. Marañón, 44-50, Barcelona, Catalonia, 08028 Spain; 6 Centre de Recerca Matemàtica, Edifici C, Campus de Bellaterra, Cerdanyola del Vallès, Catalonia, 08193 Spain

**Keywords:** Biophysical neuron models, Artificial neural networks, Wavelet transforms, Wavenet, Identification

## Abstract

Mathematical modeling of neuronal dynamics has experienced a fast growth in the last decades thanks to the biophysical formalism introduced by Hodgkin and Huxley in the 1950s. Other types of models (for instance, integrate and fire models), although less realistic, have also contributed to understand neuronal dynamics. However, there is still a vast volume of data that have not been associated with a mathematical model, mainly because data are acquired more rapidly than they can be analyzed or because it is difficult to analyze (for instance, if the number of ionic channels involved is huge). Therefore, developing new methodologies to obtain mathematical or computational models associated with data (even without previous knowledge of the source) can be helpful to make future predictions. Here, we explore the capability of a wavelet neural network to identify neuronal (single-cell) dynamics. We present an optimized computational scheme that trains the ANN with biologically plausible input currents. We obtain successful identification for data generated from four different neuron models when using all variables as inputs of the network. We also show that the empiric model obtained is able to generalize and predict the neuronal dynamics generated by variable input currents different from those used to train the artificial network. In the more realistic situation of using only the voltage and the injected current as input data to train the network, we lose predictive ability but, for low-dimensional models, the results are still satisfactory. We understand our contribution as a first step toward obtaining empiric models from experimental voltage traces.

## Introduction

Modeling based on biophysics or statistical mechanics has allowed big advances in Neuroscience. Contributions like Lapicque’s (1907) introducing the integrate-and-fire formalism to model neuron’s activity or the conductance-based formalism introduced by Hodgkin and Huxley (1952) together with a myriad of experimental work have led to a corpus of specific models that is extremely useful to model and make predictions in Neuroscience. Advances in single-cell electrophysiology have emerged through ingenious procedures often based on the application of (controlled) current injections while recording the only measurable variable, the membrane voltage. They have made it possible to identify the active ionic channels, measure its ionic conductances and, ultimately, manage to describe channel dynamics. Modeling work has benefited from these advances and has focused primarily on single-cell studies. In the realm of neural networks, experimental techniques (magnetic resonances, diffusion tensor imaging, calcium imaging, magnetoencephalography, multielectrode recordings, local field potentials,...), as well as modeling approaches, have made a great progress in recent decades. However, the insight into neuronal population dynamics has progressed more slowly, mainly because the complexity of neuronal networks (different cell types, connectivity configurations, spatiotemporal scales,...) does not allow a biophysical comprehensive approach as for the single-cell studies. Population models have been basically developed using mean-field approaches that assume a high degree of homogeneity, see for instance the seminal papers of Knight (1972) and Wilson and Cowan (1972). Examples of different approaches to model network dynamics are firing rate models (Wilson and Cowan 1972; Chizhov et al. 2007; Montbrió et al. 2015), population density models (Knight et al. 1996; Brunel and Hakim 1999; Nykamp and Tranchina 2000; Apfaltrer et al. 2006; Ly and Tranchina 2007; Chizhov and Graham 2007), neural mass models (Freeman 1972, 1975), neural fields (Amari 1977; Beim Graben and Rodrigues 2013), kinetic theory (Ventriglia 1974; Knight et al. 2000) and graph theory (Sporns 2010).

Despite this humongous work, there is still a vast volume of data that have not been associated with a mathematical model, due to different reasons, specially recordings that have not been followed by a meticulous electrophysiological task to uncover the channel dynamics (because of costs, other goals, or physical constraints) or data obtained from indirect registering methods (e.g., non-invasive). Thus, developing new methodologies to obtain mathematical or computational models associated with data (even without much prior biophysical information) can be helpful to make predictions in future experiments.

*Artificial neural networks* (ANNs) were born as replicas of brain circuitry (McCulloch and Pitts 1943; Rosenblatt 1962) and evolved along scientific paths that were practically disconnected from their inspirational biological counterpart. Nevertheless, in recent years, we have been increasingly witnessing connections between machine learning and neuroscience, in both directions, see Saxe et al. (2021). However, as far as we know, few contributions have addressed the problem of using artificial neural networks as *black boxes* that potentially substitute biophysical models.

The main goal of this paper is to prove the ability of an artificial network to identify the neural dynamics of a model and make predictions. We aim to provide an in silico proof of concept in order to apply the same procedure to experimental data later. More precisely, taking as input the time traces of the system variables in response to a prescribed injected current, we train the neural network on this input data and then use the trained network to predict the voltage response to other injected currents. In this paper, we perform this procedure in two paradigms: using all variables as input, i.e., voltage and auxiliary variables (we will call it *Paradigm I*), and using only voltage (*Paradigm II*). Note that Paradigm I aims to address the fundamental goal of the paper (the ability of the ANN in learning neuronal data), while Paradigm II is essential to our ultimate goal of applying the methodology to experimental data because in the experiments only the voltage trace can be measured.

In particular, the input current applied to a specific mathematical neuron model is designed to sweep the biophysically reasonable range, according to the corresponding bifurcation diagram. In this way, the ANN can “learn" about all the physiologically plausible neuron’s responses. The input current and the time course of the state variables obtained from the simulations constitute the inputs that feed the network. The training procedure allows us to identify the ANN parameters that fit the best to the data, and ultimately, it provides a black-box model of the neuron that can be used as a predictive or inference tool. The mathematical models simulated in this paper have been chosen to incorporate complexity gradually and illustrate the effect of the increase in dimensionality and the appearance of new time scales. The choice of parameters for each model has been made so that the bifurcation diagram (for constant input currents) presents only quiescent states and regular spiking, which are the simplest dynamic behaviors; however, as a result of applying non-constant input currents, we can eventually induce bursting-like or other complex behaviors.

In this paper, we choose a specific type of ANN based on wavelets, which we will refer to as *wavenet*, in which activation functions are based on Mallat’s multiresolution analysis (Mallat 1989). Although we could achieve similar results with other types of ANNs, wavenets have the advantage that lead to solve a linear optimization problem which provides a global solution.

The paper is organized as follows: In Sect. 2, we present the methods used along the paper, namely the structure of the artificial neural network (*wavenet*) and the training-and-testing procedure. Section 3 is devoted to the presentation of the results, which are finally summarized in Sect. 4 and discussed in Sect. 5. In order to ease the reading of the article, the neuron models used to obtain the data are presented in “Appendix A”.

## Methods

In order to test the modeling capability of our artificial neural network, we first simulate the neuron models described in “Appendix A” using as inputs those described in Sect. 2.1.4. The ensemble of inputs and outputs involved in these simulations is then combined to build up the dataset that we use to train the network for each neuron model. Section 2.1 is devoted to explain the specific artificial neural network used in this paper (that is, what we call *wavenet*); we give details of how we train and test the network in Sect. 2.1.6. Finally, in Sect. 2.1.5 we also present the methodology used to choose the hyperparameters of the network, a key point for the computational performance of our methods. Computational details of the neural network are provided in Sect. 2.2.

### Artificial neural network

Artificial neural networks consist of nodes in different layers (usually an input layer, an output layer and one or more hidden layers), interconnected with weights resulting in high-dimensional and mathematically complex models that are capable of predicting a large variety of processes. The most used neural network for control and nonlinear system’s identification is the feed forward, which profits from the *backpropagation* algorithm during supervised training (Rumelhart and McClelland 1986).

However, due to the complexity of the network structure (number of hidden layers, nodes per layer, activation functions, etc.) and training parameters (weights, loss function, etc.) that one has to heuristically set up (Haykin 1999), feed-forward networks are mathematically less tractable and, sometimes, they are replaced by nonlinear models that are linear on their parameters, so that the problem *reduces* to determine the parameters that approximate the underlying data in a given functional space. *Wavenets* (networks composed by wavelets) belong to this second kind of model: they consist of a single hidden layer where each artificial neuron (also called *node*) has a different activation function. A pair formed by a node and its associated activation function is called a *wavelon*. The activation functions are based on Mallat’s multiresolution analysis (Mallat 1989) which provides a structure for the approximation of functions at different degrees of resolution, based on wavelets theory (Daubechies 1992; Strang and Nguyen 1996), see Sect. 2.1.2 for details. Therefore, the *approximation function* for which we seek is a linear combination of the activation functions and the training process consists in determining the coefficients of the projection of the function underlying the data over the different frames of the multiresolution subspaces; these coefficients will be the weights of the wavenet.

The values of the weights of the network are obtained by solving a low-rank system of linear equations using the least-squares method (see Sect. 2.1.1). Therefore, in the context of this paper, *training the network* will simply mean *computing the set of coefficients of a frame*. We next explain the specific network structure of our wavenet and the training process in more detail. In this paper, the input dataset will always come from values of the state variables (generally, the membrane voltage *v* of the cell and other auxiliary variables with biophysical meaning) and input current values. The output variables will always coincide with the state variables.

#### Wavenet structure and training

Our goal is training the network in order to identify every system presented in “Appendix A”, that is, obtaining a wavenet that mimics the dynamics of each neuron model. Sometimes, we will refer to this procedure as the *identification of the neuron*.

In order to succeed in the training process, there have to be enough variations in the inputs so that most of the possible situations in the estimation phase are embedded in the wavenet model. For this purpose, we will use the current $I_{app}$ (see Sect. 2.1.4) that appears in all the models as a control input that will allow the numerical simulation to visit a wide range of dynamical states of the system.

**Fig. 1:**
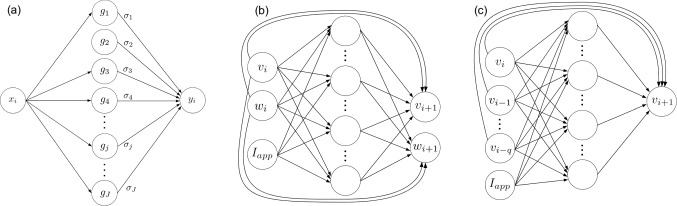
Wavenet structure and training. **a** Simplified diagram of a single variable (1-input) wavenet. **b** Simplified wavenet representation for the prediction of a two-dimensional model with an external stimulus $I_{app}$ in Paradigm I: we feed the network with the values of all the state variables and the input current at time *i* (input nodes; left); the information flows through the wavenet and gives the predicted values of all the state variables (output nodes; right). The long-range connections stand for the linear terms added to the wavenet structure. **c** Same as **b** for Paradigm II, that is: we feed the network with $q+1$ values of the voltage variable and the input current at time *i* and it gives the predicted value of the voltage variable

Depending on the paradigm, we will have different inputs and outputs of the network:

I In *Paradigm I*, for each time step, we will have the current values of the state variables plus the value of the control input current as inputs of the network, while the output will be the values of the state variables at the following time step, obtained from numerical integration; see Fig. 1b for a schematic picture in the case of two state variables *v* and *w*.
II In *Paradigm II* (closer to the experimental situation), for each time step, the inputs of the network will be the current voltage sample and the *q* preceding ones (*q* to be determined for each model) plus the value of the control input current, while the output will be the values of the voltage variable at the following time step, obtained from numerical integration; see Fig. 1c for a schematic picture.

We will denote by ${\varvec{x}}=\{x^{(m)}\}^{N_E}_{m=1}$ the *input dataset* obtained from simulating a neuron model during $N_E$ integration steps. In the example of Paradigm I shown in Fig. 1b, $x^{(m)}=(v(t_{m-1}),w(t_{m-1}),I_{app}(t_{m-1}))$, where $t_{m-1}$ is the time at which we apply the *m*-th integration step. In the example of Paradigm II shown in Fig. 1c, $x^{(m)}=(v(t_{m-1}),\dots ,v(t_{m-1-q}),I_{app}(t_{m-1}))$. The *output dataset* will be denoted by ${\varvec{y}}=\{y^{(m)}\}^{N_E}_{m=1}$; in the example of Fig. 1b, $y^{(m)}=\left( v(t_m),w(t_m)\right)$ and in the example of Fig. 1c, $y^{(m)}=v(t_m)$.

In order to explain the general approximation procedure, we will momentarily consider $x^{(m)}$ to be one-dimensional and assume that we want to approximate a one-variable real function *f* by a wavenet formed by *J* nodes, which correspond to the number of nodes in the middle layer of the panels of Fig. 1a. Let ${\varvec{g}}=(g_1,\dots ,g_J)$ be the vector of the corresponding activation functions, with $g_j$ belonging to a frame of functions within a function space that approximates *f* to a given bounding error, see Sect. 2.1.2. Note that, with this notation, the wavelons are the pairs $(j,g_j)$, for $j=1,\dots ,J$. Thus, the approximation procedure builds upon

1 $$\begin{aligned} f(x^{(m)}) = {\varvec{g}}(x^{(m)})\,{\varvec{\sigma }}, \end{aligned}$$

for $m=1,\dots ,N_E$, where $f(x^{(m)})=y^{(m)}$, ${\varvec{g}}(x^{(m)})$ is a row vector of dimension *J* and ${\varvec{\sigma }}$ is a column vector of dimension *J* whose components correspond to the values of the projection of *f*(*x*) over each frame function, that is, the weights of the network.

In a compact way, we can write equation (1) as the low-rank system:

2 $$\begin{aligned} {\varvec{{\hat{y}}}} = {\varvec{G}}\, {\varvec{\sigma }}, \end{aligned}$$

where ${\varvec{{\hat{y}}}}$ is a column vector of dimension $N_E$ where each component is a different output $y^{(m)}:=f(x^{(m)})$, and ${\varvec{G}}$ is a $N_E\times J$ matrix where each row is given by ${\varvec{g}}(x^{(m)})$. In this way, training the wavenet consists of determining ${\varvec{\sigma }}$, the vector of the coefficients, by solving system (2).

To solve the low-rank linear system in equation (2), the mean-squared error (MSE) is used as an objective function, following the multiple linear regression expression presented in Claumann (2003):

3 $$\begin{aligned} MSE = \frac{1}{N_E}\Vert {\varvec{y}}-{\varvec{G}}{\varvec{\sigma }} \Vert ^{2}. \end{aligned}$$

Later on, the norm vector of the squared weights ${\Vert \sigma \Vert }^{2}$ is added as a *regularizer*, turning the MSE function into a *regularized objective function*. The regularizer is able to smooth the curvature of the model’s surface by reducing the oscillations around the data used as a target for the model and avoiding possible problems related to the inverse of the covariance matrix when solving the minimized system with the least-squares method. The new objective function $\mathcal {O}$ is

4 $$\begin{aligned} \mathcal {O}=\frac{1}{N_E} \left( \Vert {\varvec{y}}-{\varvec{G}}{\varvec{\sigma }} \Vert ^{2}+\gamma \Vert {\varvec{\sigma }}^{2} \Vert \right) . \end{aligned}$$

The $\gamma$ parameter is a multiplier; indeed, $\gamma = \mu \lambda ^{max}$, where $\lambda ^{max}$ is the largest eigenvalue of the covariance matrix ${\varvec{G}}^\intercal {\varvec{G}}$ and $\mu$ is a constant found through experimentation. In order to minimize the objective function, that is, $\frac{\partial \mathcal {O}}{\partial {\varvec{\sigma }}} = 0$, we must solve the linear system

5 $$\begin{aligned} ({\varvec{G}}^\intercal {\varvec{G}}+\gamma I ){\varvec{\sigma }} = {\varvec{G}}^\intercal {\varvec{{\hat{y}}}}. \end{aligned}$$

**Fig. 2:**
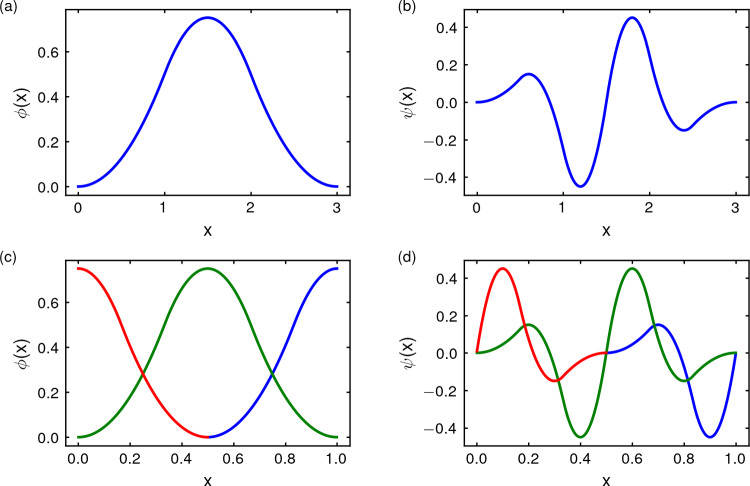
Scale function and displaced scaling functions. Scale function **a** and three displaced scaling functions **c** associated to it. Here, we use the so-called *quadratic spline* scaling function, see (7); similar representations are achieved from other choices of scaling functions. The functions $\phi _0$ (in blue), $\phi _1$ (in green) and $\phi _2$ (in red) are computed from (8) with $d=N_S=3$. In the rightmost panels, we show the corresponding wavelet mother (**b**) and its displaced wavelets (**d**), $\psi _0$ (in blue), $\psi _1$ (in green) and $\psi _2$ (in red), which are computed from (9). All functions are already restricted to the support $\Omega =[0,1]$

#### Activation functions

We defined the activation functions associated with the network nodes from Mallat’s multiresolution analysis (see Mallat (1989) or Mallat (2008), Ch. 7) with additional modifications introduced for computational purposes. Our particular *frame of functions* is constructed upon a *scaling function*. In general, scaling functions are defined as:

6 $$\begin{aligned} \phi (x) = \sum _{n = 0}^N p_n \phi (2x - n), \end{aligned}$$

where $p_n$ are called *interscale coefficients* and fulfill $\sum _{n = 0}^N p_n = 2$, see Strang (1989) and Claumann (2003). In this paper, we have used standard scaling functions; as a particular example, in Fig. 2, we show the quadratic spline scaling function, given by

7 $$\begin{aligned} \phi (x)= \left\{ \begin{array}{ll} \frac{x^2}{2} &{} x\in [0,1),\\ -x^2+3\,x-\frac{3}{2} &{} x\in [1,2),\\ \frac{x^2}{2}-3\,x+\frac{9}{2} &{} x\in [2,3],\\ 0 &{} \text{ otherwise, } \end{array} \right. \end{aligned}$$

and rescaled to the interval [0, 1]. In general, we assume that the scaling functions have compact support on an interval $\Omega =[0,d]$. The frame of functions that we consider consists of three groups of functions:

1. A set $\{\phi _k\}_{k=0}^{N_S-1}$ of *displaced scaling functions*, obtained by translations of the scaling function $\phi$, see Fig. 2. Each function $\phi _k$ is centered at $x=1-\frac{k}{N_S-1}$. The parameter $N_S>1$ is chosen *ad hoc* in each example. The precise formula, for $k=0,\dots ,N_S-1$, to obtain these displaced functions is:
  8 $$\begin{aligned} \phi _k(x):= {\left\{ \begin{array}{ll} \phi \left( \left( x-\frac{1}{2}+\frac{k}{N_S-1}\right) d\right) &{} \ x\in [0,d],\\ 0 &{} \text{ otherwise }. \end{array}\right. } \end{aligned}$$
2. The family of *wavelet functions* $\{\psi ^{(k)}_{r,n}\}$, with $(r,n)\in {{\mathbb {Z}}}^2$, corresponding to each $\phi _k$, for $k=0,\dots ,N_S-1$. Each family is obtained from the corresponding mother wavelet
  9 $$\begin{aligned} \psi _k (x) = \sum _{n = 0}^N (-1)^n p_n \phi _k(2x - n), \end{aligned}$$
  and each wavelet is defined as
  10 $$\begin{aligned} \psi ^{(k)}_{r,n} (x) = |a_0|^{r/2} \psi _k (a_0^r x - n b_0), \ r,n \in {\mathbb {Z}}, \end{aligned}$$
  where $a_0 = 2$ and $b_0 = 1$ have been chosen, as in Claumann (2003). The parameter *r* is called the *resolution level* and $n \in \{0,1, 2,\ldots , 2^r -1\}$ represents the so-called *translation index*.
3. We also consider the identity function of each state variable. As proposed in Fisco-Compte (2020) and Alexandridis and Zapranis (2011), it might be useful to reduce the prediction error. This is equivalent to approximating $f(x)-L(x)$, where *L*(*x*) is a linear function of the state input vector, instead of *f*(*x*) in Eq. (1), by the neural network. This corresponds to adding new edges to the wavenet; more precisely, the peripheral connections in Fig. 1b. For the sake of simplicity, we will consider that each pair formed by a node containing a variable of the system and the identity function is also a wavelon, despite the fact that the function is not a wavelet.

Finally, all functions of the frame are restricted to $\Omega$ and taken as zero on $\Omega ^c$. In order to avoid a cumbersome notation, we do not change the names of the functions after this restriction. We will call ${{\mathcal {F}}}$ the resulting frame of functions:

11 $$\begin{aligned} {\mathcal {F}}:=\textrm{Id}\cup \{\phi _k\}\cup \{\psi ^{(k)}_{r,n}\}, \end{aligned}$$

where $$k=0,\dots ,N_S-1$$; $$r=0,\dots ,N_R-1$$ and $$n=0,\dots ,2^r-1$$.

In the practical implementation, we are seeking for an approximation

12 $$\begin{aligned} \begin{aligned} {\hat{f}}(x)&= \ a\,x + \sum _{k=0}^{N_S-1} b_k \phi _k(x) \\&\quad + \sum _{k=0}^{N_S-1} \sum _{r=0}^{N_R-1} \sum _{n = 0}^{2^r-1} c^{(k)}_{r,n}\psi ^{(k)}_{r,n}(x), \end{aligned} \end{aligned}$$

where we will have to determine the parameters *a*, $b_k$, $c^{(k)}_{r,n}$, $N_S$ (number of displaced scaling functions) and $N_R$ (levels of resolution). The higher the level of resolution one achieves, the finer the approximation of the function. However, more levels of resolution mean larger vector functions ${\varvec{g}}$ which results in a higher computational cost when solving system (2) by the modified least-squares method. Notice that ${{\mathcal {F}}}$ adds redundancy to the Mallat’s scheme because we increase the number of functions of the frame. Despite losing the structure of basis, we gain capability to capture the details of the system. This strategy increases the computational effort in each resolution level but lowers the number of levels needed to reach the same performance, thus reducing the overall computational costs, see also Claumann (2003).

Apart from the exponential increase in wavelons when the number of independent variables increases, wavenets also present some issues in terms of domain support. Scaling functions usually take values close to zero at both ends of their domain which results in data points closer to the center of the domain having a greater relevance in the regression problem. As a consequence, prediction of data points that live in both ends of the activation function domain is less reliable. The superposition of the displaced scaling functions (8) in the same domain (Claumann 2003), which in turn results in superposition of wavelets in higher resolution levels, mitigates this problem. The number of wavelons increases by a factor of $N_S^{N_I}$ but, as pointed out above, the improved performance allows for fewer levels of resolution in order to obtain accurate results.

Note that the components of the vector function ${\varvec{g}}$ considered above are the functions belonging to ${{\mathcal {F}}}$. In a *wavenet* diagram, as in Fig. 1b, the addition of the identity function is represented by a direct connection between the input and the output, without an intermediate node. All other functions correspond (one-to-one) to the nodes of the middle layer.

#### The multi-input/multioutput case

The procedure explained so far for a single input and a single output can be naturally extended both to the multi-input, by interpreting each vector input as a single input, and to the multioutput case, by considering a specific problem for each output component. However, as we will explain in detail, the number of functions of the frame up to a certain level of resolution increases exponentially with the dimension of the input, and so does the number *J* of wavelons in the wavenet. For instance, in the example shown in Fig. 1b, the dimension of the input is $N_I=3$ since $x=(v,w,I_{app})$ while the dimension of the output is $N_O=2$ since $y=(v,w)$.

While, for the single-input case, the levels of resolution are easily defined from the scaling functions, for the multi-input case, the definition is more elaborate, see Claumann (2003) and Fisco-Compte (2020).

The level of scaling functions is composed by $N_S^{N_I}$ functions defined as all the combinations of products of the $N_S$ scaling functions applied to the $N_I$ input variables; that is, $N_I$-variable functions of type

13 $$\begin{aligned} \phi _{i_1}(x_1)\phi _{i_2}(x_2)\cdots \phi _{i_{N_I}}(x_{N_I}), \end{aligned}$$

for $i_j\in \{0,\dots ,N_S-1\}$. For instance, in the example shown in Fig. 1b, we have 27 functions in this level (assuming that $N_S=3$).

The first level of resolution of wavelets represents the $r=0$ level of resolution. It involves products of mother wavelets $\psi ^{(k)}_{0,0}$, together with scaling functions $\phi _k$, for $k=0,\dots ,N_S$; that is, $N_I$-variable functions of type

14 $$\begin{aligned} \xi _{i_1}(x_1)\xi _{i_2}(x_2)\cdots \xi _{i_{N_I}}(x_{N_I}), \end{aligned}$$

for $i_j\in \{0,\dots ,N_S-1\}$, where $\xi _{*}$ represents either a scaling function or a mother wavelet, excluding the case in which all $\xi _{i_j}$, for $j=1,\dots ,N_I$, are scaling functions, which has already been considered in (13). Note that this leads to $(2\,N_S)^{N_I}-N_S^{N_I}$ functions. For instance, for $N_I=N_S=3$, we get 189 functions in this level.

The following levels of resolution are constructed in a similar way than (14) but taking $\xi _{*}$ as either scaling functions or wavelets of the corresponding level of resolution as defined in (10), always excluding the case in which all $\xi _{i_j}$, for $j=1,\dots ,N_I$, are scaling functions. Note that for $r\ge 1$, we are considering $2^r$ displaced scaling functions and so the number of functions grows rapidly with *r*. For instance, if we have $N_I=3$ input variables (e.g., Morris–Lecar model or Fitzhugh–Nagumo model, see “Appendix A”), the number of functions at the level of resolution *r* is $N_S^3\left( 3\cdot 2^{r} + 3\cdot 2^{2r} + 2^{3r}\right)$; for $N_S=3$ $(N_S=4)$, this gives 702 (1664), 3348 (7936), 19656 (46592), 132624 (314368),...functions for $r=1,2,3,4,\dots$, respectively. Fortunately, for the purposes of this paper (from Sect. 3.1 on), we get satisfactory results with only few levels of resolution.

Finally, it is also possible to approximate vector (i.e., multioutput) functions

15 $$\begin{aligned} {\varvec{f}}({\varvec{x}}) = \left( f_1({\varvec{x}}),\dots , f_n({\varvec{x}})\right) . \end{aligned}$$

by applying the above procedure [that is, solving system (2)] for each component of ${\varvec{f}}({\varvec{x}})$. Note that, since the input data are the same for each component, all *n* systems share the matrix ${\varvec{G}}$ and one only needs to change the output vector ${\varvec{{\hat{y}}}}$ in (2) by $\{f_i({\varvec{x}}^{(m)})\}^{N_E}_{m=1}$.

#### Current inputs for training

**Fig. 3:**
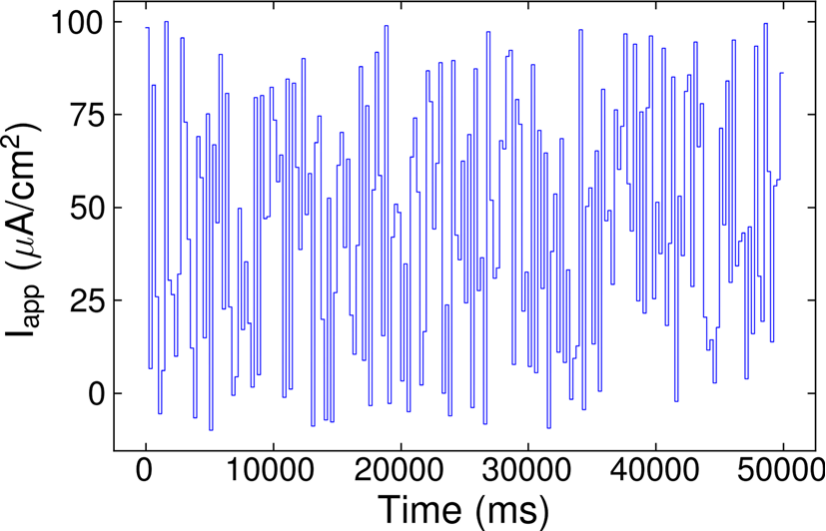
Input current used for training. Trace of the $I_{app}$ signal used to train the Morris–Lecar model (see “Appendix A.1”)

Input currents used for training are represented in the models by the $I_{app}$ term. For this purpose, $I_{app}(t)$ is generated randomly, in a way that forces the system to visit all biologically plausible regimes of the neuron and causes all possible dynamical states to manifest. For any model, the *biologically plausible regimes* are determined from the corresponding bifurcation diagram (see Figs. 20, 21, 22 and 23, respectively) by sampling the $I_{app}$ within a prescribed interval ${{\mathcal {I}}}$ close to the bifurcation between equilibria and limit cycle oscillations. We want to cover both equilibrium states at any level of excitability within the physiological range and oscillations at any physiological frequency. For values out of this interval, the current values are either so small that the cell remains hopelessly in a quiescent state or too large that they can induce a nerve block. Moreover, the current $I_{app}(t)$ has to be designed so that enough time ($\Delta T$) is spent in each equilibrium or oscillation value for the network to learn; that is, we want the wavenet to account for all possible responses of the neuron model to external excitation. This results in a stepwise temporal series that takes uniformly distributed random values in ${{\mathcal {I}}}$. In Fig. 3, a sample of an $I_{app}$ signal used to identify the Morris–Lecar model (see also Fig. 20) is shown. As it can be seen, the signal remains a certain period of time in a constant $I_{app}$ value, allowing the model to reach the stationary state in its trajectory with the objective of obtaining a rich dataset that is suitable for training the wavenet.

The interval ${{\mathcal {I}}}$, the parameter $\Delta T$, and the training duration are selected for each model and experiment. In particular, ${{\mathcal {I}}}=[20, 60]$ for the Morris–Lecar model, [0.07, 0.09] for the FitzHugh–Nagumo model, [0.05, 0.07] for the FitzHugh–Nagumo–Rinzel model and $[-3,3]$ for the Wang model. Because they were less computationally demanding, the training for the 2D models lasted 2500 s but it could be much less; for the Fitzhugh–Nagumo–Rinzel model it lasted 375 s and for the Wang model, 230 s.

#### Wavenet’s hyperparameters selection

Hyperparameters are the parameters that are set before the learning process is started, such as the number of superposed wavelets discussed at the end of Sect. 2.1.1, the number of resolution levels, the scaling functions used as activation functions (see Sect. 2.1.2) and the regularizer (a parameter that improves training results and computational robustness), defined right after (4).

Hyperparameters are determined previous to training with the purpose of optimizing overall performance of the network, aiming at reducing both the prediction error and the computation time. With the aim of helping the user make an appropriate choice of hyperparameters, we have performed a prediction error versus computational cost study in which we explore many of the possible hyperparameter combinations.

The regularizer is studied apart from the other hyperparameters. The $\mu$ parameter from the regularizer will be set to a constant value of $2\cdot 10^{-16}$ during the study so as not to interfere with the mathematical features of the model; its main function is to introduce a small computational error to avoid numerical problems with the matrix inverse while the least-squares method is applied, as mentioned in Sect. 2.1.1.

We use the FitzHugh–Nagumo model, see Sect. A.2, as a showcase. We study the behavior of the hyperparameters of the corresponding wavenet and propose possible configurations when training this specific model. The wavenet has been trained to approximate the FitzHugh–Nagumo model for each combination of hyperparameters, keeping the same set of inputs, and the testing scores have been stored and compared. The training dataset is obtained by integrating equations in (A.4) with a time step of $\Delta t = 0.05$ ms, with 500 different random $I_{app}$ values, drawn from a uniform distribution in the interval ${\mathcal I}=[0,0.1]$ and kept constant for $\Delta T=200$ ms. Note that this specific choice of $\Delta T$ and the number of $I_{app}$ values is only used here, for the Fitzhugh–Nagumo model, in order to select the hyperparameters of the network. Thus, for each hyperparameter configuration, we integrate over 100 s. In the testing simulations, we use shorter simulations.

The study of hyperparameters is organized along the following steps:

1. A plausible range for each hyperparameter is selected. We have chosen $N_R= 0, 1, 2$ for a total of three different levels of resolution, $N_S= 4$ and 5 superposed activation functions in the wavenets, as discussed in the last paragraph of Sect. 2.1, and two different scaling functions: the quadratic spline and the bicubic spline.
2. The model is trained with one of the 12 possible combinations at a time.
3. The total number of wavelons used to approximate the neuron model and the relative error of the prediction are used as scores. The error is computed using a test dataset to assess the robustness of the prediction during 5000 ms of the oscillatory signal used for this model (see Table 2). The error score taken is from the *v* variable of the FitzHugh–Nagumo model.
4. The best performing hyperparameters are chosen according to a balance between the computational cost, defined by the number of wavelons, and the prediction error. As the number of wavelons is a measure of the wavenet complexity, it is also an estimator of the time needed to train the neural network.

#### Testing wavenet’s generalization: indicators

Once the network has been trained, we get a solution ${\varvec{{\hat{\sigma }}}}$ of the least-squares problem (5) associated with system (2). In order to test the training process and assess the ability of the network to generalize, we consider a test dataset, $\left( {\varvec{x_T}},{\varvec{y_T}}\right)$, which is obtained by integrating the ODEs of the neuron model subject to a new perturbation $I_{app,T}(t)$ for $N_T$ integration steps with initial conditions at times $\{t_m\}^{N_T-1}_{m=0}$. Thus, as in Sect. 2.1.1, ${\varvec{x_T}}=\{x_T^{(m)}\}_{m=1,\dots ,N_T}$ and ${\varvec{y_T}}=\{y_T^{(m)}\}_{m=1,\dots ,N_T}$, where $x_T^{(m)}$ has $N_I=n+1$ components, being *n* the number of state variables, and $y_T^{(m)}$ has $N_O=n$ components. The components $x_T^{(m)}$ are the values of the *n* state variables at time $t_{m-1}$ and the value of $I_{app,T}$, whereas the components $y_T^{(m)}$ are the values of the *n* state variables at time $t_{m}$. We then evaluate ${\varvec{G_T}}:={\varvec{G}}({\varvec{x_T}})$ and compute ${\varvec{{\widehat{y}}_T}}:={\varvec{G_T}}\,{\varvec{{\hat{\sigma }}}}$.

In order to assess the accuracy and validity of the wavenet predictive capabilities, we have compared the test and the predicted output datasets, ${\varvec{y_T}}$ and ${\varvec{{\widehat{y}}_T}}$, respectively, using different indicators:

- The $(1 - r^2)$*-score*: For the *i*-th state variable, for $i=1,\dots ,n$, we define
  16 $$\begin{aligned} (1 - r^2)_i = \frac{\sum _{m=1}^{N_T}\left( y_T^{(m)}-{\widehat{y}}_T^{(m)}\right) ^{2}}{\sum _{m=1}^{N_T}\left( y_T^{(m)}-{\overline{y}}_T\right) ^{2}}, \end{aligned}$$
  where $\overline{y}_T$ is the mean of the variable in the test dataset. The closer the $(1-r^2)_i$ error gets to zero, the better the prediction for the *i*th state variable.
- The *normalized cross-correlation function* between the test and the predicted output datasets is given by:
  17 $$\begin{aligned} R(k):= \frac{\sum ^{N_T}_{m=1}y_T^{(m)} \, \widehat{y}_T^{(m+k)}}{\Vert y_T \Vert \, \Vert \widehat{y}_T \Vert }, \end{aligned}$$
  where *k* is the displacement or lag.
- The *cosine similarity*: the cosine of the angle between the projection and the original signal,
  18 $$\begin{aligned} S_C:=S_C(\widehat{y},y)= R(0) \end{aligned}$$

It is worth mentioning that the $(1 - r^2)$-score provides a good detection of the outstanding identifications but, in the presence of time-lags in the predicted signal, it can be a pessimistic indicator, even if the spikes are all well identified. As we will see in the results, in these cases, the cross-correlation *R* and the cosine similarity $S_C$ provide a better assessment of the goodness of the identification. For this purpose, we will also compare the distributions of *interspike intervals* (ISI) of *y* and $\widehat{y}$ to illustrate how well essential features like the number of spikes and the frequency are predicted (given a sequence of spike times $\{t_1,\dots ,t_p\}$ of a specific voltage time series, with $t_1<t_2<\dots <t_p$, we define $\text{ ISI}_j=t_{j+1}-t_{j}$, for $j=1,\dots ,p-1$).

#### Robustness of the identification method

In the testing phase, our aim is to assess the robustness of the identification method. On the one hand, we will consider noise in some parameters of the system and, on the other, we will introduce different random features in the currents used for training.

Models are modified by adding uniformly distributed noise to some of its relevant parameters. More, precisely, for a given parameter, say $\kappa$, we consider $\kappa (1+\xi (t))$, where $\xi (t)$ is randomly distributed in $[-{\bar{\xi }},{\bar{\xi }}]$, for some ${\bar{\xi }}={\bar{\xi }}(\kappa )>0$. For the conductance-based models, that is, the Morris–Lecar (“Appendix A.1”) and Wang models (“Appendix A.4”), the noisy parameters are the leakage conductance, $g_L$, and the potassium conductance, $g_K$, while for the FitzHugh–Nagumo models (“Appendices A.2 and A.3”), the noisy parameters are *a* and $\gamma$. In our simulations, we have taken ${\bar{\xi }}=0.01$ since, from a biological and an engineering standpoint, it is plausible to work with this percentage of data variation.

**Fig. 4:**
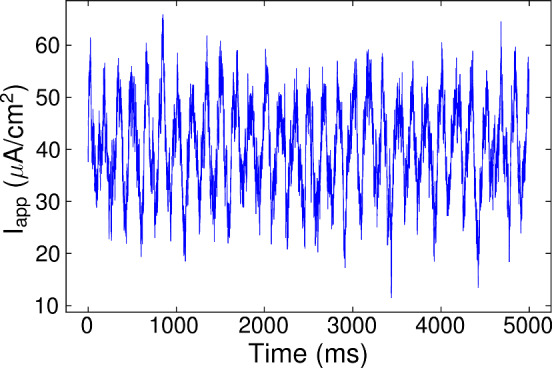
Oscillatory testing input current. Example of input data generated from the stochastic process (19) and used for predictions in the Morris–Lecar model

Two types of random testing currents have been considered:

1. *Stepwise testing current.* The stepwise input current used for testing has been generated randomly as the input currents used for training, see Fig. 3. The parameter values used to generate such input are shown in Table 1. They were also selected in order to place the neuron’s activity in the biologically plausible regime (see again Figs. 20, 21, 22 and 23, respectively).
2. *Oscillatory testing current.* In this case, the deterministic model is externally forced by means of an *oscillatory process* defined by:
  19 $$\begin{aligned} \begin{array}{rl} \dfrac{dI_{app}}{dt} =&{} \frac{1}{\tau }(I_{app,0} + \nu \cos (\omega \,t)-I_{app}) \\ &{}\\ &{}+ \sigma dW_t, \end{array} \end{aligned}$$
  where $W_t$ is the Wiener process, which is more realistic from a biological point of view. In Fig. 4, we see one realization of the stochastic oscillatory input for the Morris–Lecar model. The input signal in the other models is adjusted accordingly to the parameters in Table 2. As with the stepwise current traces, they have been selected for the purpose of studying the neuron models around their bifurcation point (encompassing both quiescent and oscillatory regimes).

**Table 1:** Parameters of the stepwise testing current: $\# I_{app}$ refers to the number of different $I_{app}$ (random) values used to designed the testing input, $\Delta T$ is the time (in ms) that each $I_{app}$ value is kept constant and ${{\mathcal {I}}}$ is the interval of $I_{app}$ values (in ${\upmu } A/\text{cm}^2$ for Morris–Lecar and Wang models) in which the $I_{app}$ are randomly chosen

Model	$\# I_{app}$	$\Delta T$	${{\mathcal {I}}}$
Morris Lecar	50	100	[20, 60]
FitzHugh–Nagumo	50	100	[0.07, 0.09]
FitzHugh–Nagumo 3D	50	100	[0.05, 0.07]
Wang	50	100	[− 3, 3]

**Table 2:** Parameter values used in (19) to generate the oscillatory input

Model	$\tau$	$\nu$	$\sigma$
Morris Lecar	10	10	9.5
FitzHugh–Nagumo	10	0.005	0.0045
FitzHugh–Nagumo 3D	10	0.005	0.0045
Wang	10	1.5	2.85

The frequency used across all models is $\omega = 1.2\cdot \pi \cdot 10^{-4}$. The intervals ${{\mathcal {I}}}$ from which we have randomly taken the values of $I_{app,0}$ are the same as in Table 1

### Computational aspects

Let us now comment some important aspects about the implementation. As a general remark concerning the four models we have studied, we note that the hyperparameter selection provides an optimized configuration for the model to be easily explored at different levels of resolution. For two-dimensional models, the identification is possible with few levels of resolution and, therefore, few wavelons; however, in three-dimensional systems the number of wavelons is more critical, since a higher frame is needed to project the details of a complex model in the wavelet space. Computationally speaking, the Wang model resulted to be even more complex than the FitzHugh–Nagumo–Rinzel: at least the second level of resolution was needed for a good model approximation. Playing with the number of superposed functions was useful to overcome the computational difficulties derived from the exponential growth of the wavelons due to the number of wavenet inputs.

It is worth mentioning that solving the overdetermined systems needed to train the network implies a great computational cost in terms of time and memory space. The matrix of activation functions built for these models can become very large: for instance, the matrix derived in the Wang model was trained with 11500 $I_{app}$ values and 20 ms of signal in each $I_{app}$, corresponding to a total of 4000 integration steps of the model using Euler’s method. Each integration step corresponds to a row of the matrix of activation functions, and every wavelon from the wavenet’s model representation corresponds to a column of this matrix. Since the Wang model used a total of 24,579 wavelons, then the total number of elements in the matrix is $1.130634\cdot 10^{12}$, which represents nearly 8.23 TB of memory in 64-bit floating-point data. This example reveals the magnitude of the arrays used in this type of problems, with the need to compute an ordinary least-squares algorithm to a matrix with this much memory.

For the sake of simplicity, the custom algorithm is coded mainly using Python, and Cython for the construction of the matrix ${\varvec{G}}$, as a useful language to write the pure C functions used to speed up the wavelet computations in Python. As we could not have access to 8.23 TB of available memory, we had to store the matrix in a Miles et al. (2020) file with the Zstandard compression (Collet and Kucherawy 2021), using a 1 TB NVMe SSD, 192 GB of RAM and a dual Intel Xeon(R) Silver 4116 CPU workstation, for parallel chunk-based computation alongside with Dask library (Rocklin 2015).

Generally, in a context of chunk-based computations, the very dimension of the chunks would be an hyperparameter to study if a low precision data format, like 8-bit floating-point, is used with the goal to achieve an accuracy similar to having used single or double-precision floating-points, reducing the complexity of the calculations and therefore the computation time (Wang et al. 2018). Due to the fact that the wavenet has only one hidden layer and there are not enough partial product accumulations, typical of deep learning models with several hidden layers, the size of the chunks is not as significant enough to have a place in the study of hyperparameters and there is not much benefit to use other data formats than a double-precision floating point as in our case.

The distributed modules from Dask did provide us out-of-core chunk-based computations, allowing us to perform the ordinary least-squares algorithm for a 8.23 TB low-rank matrix in a single computer. The distributed modules access the matrix in the computer’s hard drive and perform partial operations on smaller chunks of data that are loaded in parallel into the RAM. All files that come from Dask operations from the disk data are stored in parquet files, such us the final weights’ vector, which is a good solution to be able to continue using the libraries Numpy (Harris et al. 2020), Pandas (Wes McKinney 2010) and Scipy (Virtanen et al. 2020) libraries to perform the following operation from the parquet files. It has to be mentioned that a JIT compiler, such as Numba (Lam et al. 2015), was used with the function that generates the arrays of data for the inputs and targets of the wavenet.

## Results

Our main contribution is the identification of different neuron models alongside with the illustration of the high predictive power of our method, see Sect. 3.2 (Paradigm I) and Sect. 3.3 (Paradigm II). Before that, in Sect. 3.1, we explain how the hyperparameters’ configuration is achieved. Despite being a very technical question, we think it is relevant since it is essential to achieve accurate predictions in the model.

### Configuration of hyperparameters

**Fig. 5:**
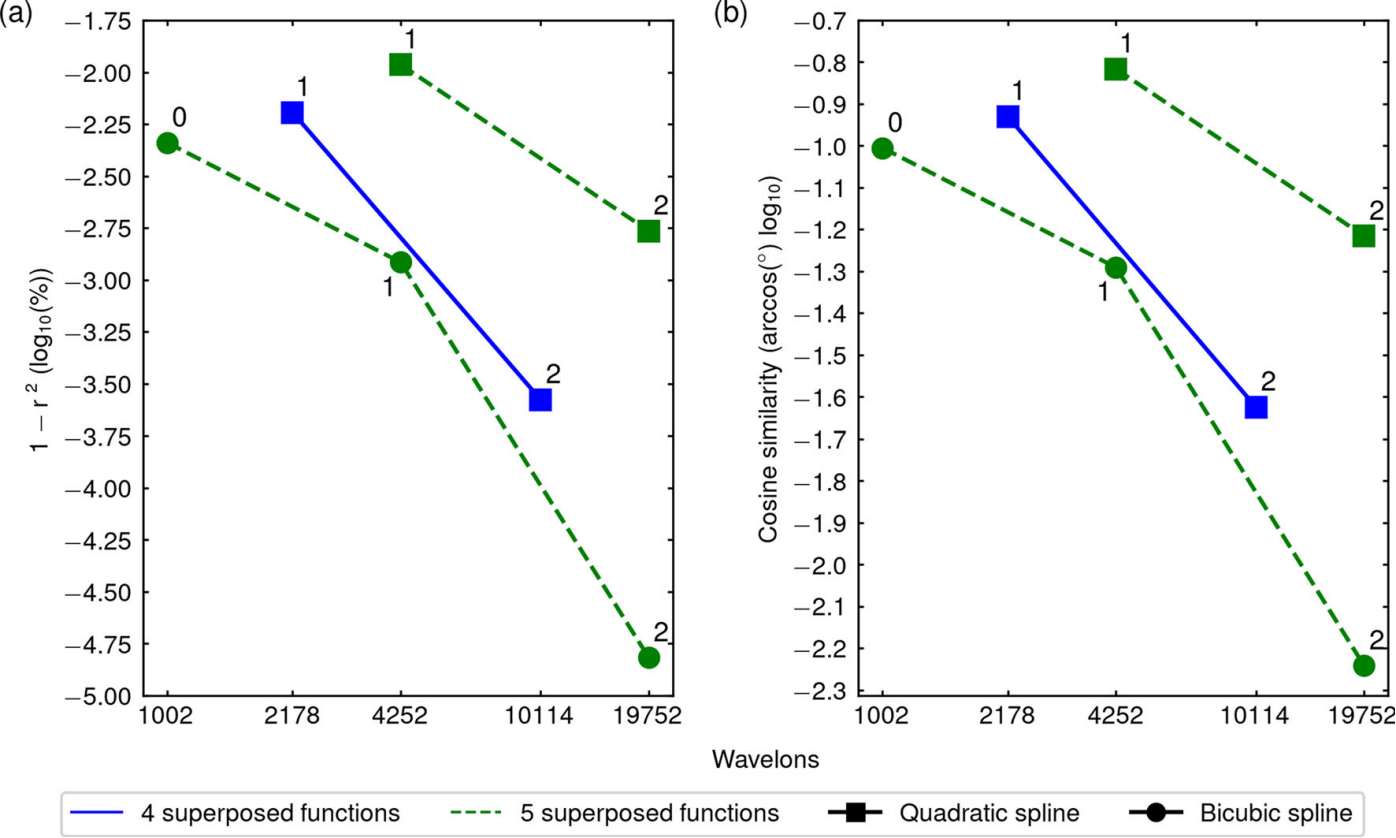
Performance of hyperparameter configurations. Performance of different wavenet hyperparameter configurations for the FitzHugh–Nagumo model (A.4) using two indicators: $(1 - r^2)$-score (**a**) and cosine similarity (**b**). In-graph numbers indicate the level of resolution ($N_R$) of the wavenet. Color encodes the number of superposed functions ($N_S$) and symbols indicate the different wavelet families

We have investigated the performance of different hyperparameter configurations of the wavenet. To choose the optimal configuration, we have taken into account not only the error of the identification but also its computational cost since, as explained in Sect. 2.1.3, the number of wavelons of the wavenet increases very fast with the levels of resolution. In Fig. 5, we show the error of different configurations used for the FitzHugh–Nagumo model (A.4) against the number of wavelons; in the left panel, the error is quantified by the $(1 - r^2)$-score (16), while in the right panel it is quantified by the cosine similarity (18). Only configurations that approximate the model with enough accuracy are displayed. Similar qualitative descriptions (not shown) were obtained for the other models, with the exception of the Wang model (A.6) that will be commented below. It is worth noticing that for three-dimensional models the number of wavelons given in the *x*-axes of Fig. 5 increases substantially.

An important factor in order to reduce the computational cost is the quality of the data. It is mainly determined by the intrinsic complexity of the model and by the choice of the $I_{app}$ inputs, which have been thoroughly chosen as explained in Sect. 2.1.4, Fig. 3 and Sect. 2.1.7.

In Fig. 5, we have explored the effect of three factors: the scaling function (encoded by point shapes in Fig. 5), the number of superposed functions ($N_S$, encoded by colors in Fig. 5), and the number of levels of resolution (*r*, indicated by numbers next to the shaped points). From the eight configurations explored, we observe:

1. The bicubic spline scaling functions with 5 superpositions give a better performance than the quadratic spline scaling functions with 4 or 5 superpositions. (The bicubic spline scaling functions with 4 superpositions is not shown because of its low performance.) In particular, the performance with bicubic splines and 5 superpositions using only up to the first level of resolution (that is, up to $r=0$ or, equivalently, $N_R=1$) is better than the performance with quadratic splines and 4 or 5 superpositions up to $r=1$, at a much lower computational expense (1002 wavelons against 2178 or 4252).
2. If we fix a scaling function and a number of superpositions so that we observe only one of the three curves, the performance (obviously) increases but the computational cost is too high. For instance, for the bicubic spline with 5 superpositions, we need 4252 wavelons instead of 1002 wavelons in order to reduce the error from $10^{-2.3}$, which is already satisfactory, to $10^{-3}$.

According to this analysis, we decided to choose the bicubic spline scaling function with 5 superpositions and $N_R=1$ (that is, up to $r=0$) for the Morris–Lecar, Fitzhugh–Nagumo and Fitzhugh–Nagumo 3D models, as displayed in Table 3. For the Wang model, the quadratic spline with 4 superposing functions (that is, the analogue to the blue curve in Fig. 5) provides the best performance compared to the other cases, but we need to go at least to the $r=1$ (that is, $N_R=2$) level of resolution to get an acceptable performance; this fact leads, unfortunately, to 24,579 wavelons, a much higher computational cost.

**Table 3:** Hyperparameters chosen for the model training: $\phi$ indicates the (spline) scaling function, $N_S$ is the number of superposed functions $N_S$ and $N_R$ is the number of levels of resolution

Model	$\phi$	$N_S$	$N_R$
Morris–Lecar	Bicubic	5	1
FitzHugh–Nagumo	Bicubic	5	1
FitzHugh–Nagumo 3D	Bicubic	5	1
Wang	Quadratic	4	2

**Fig. 6:**
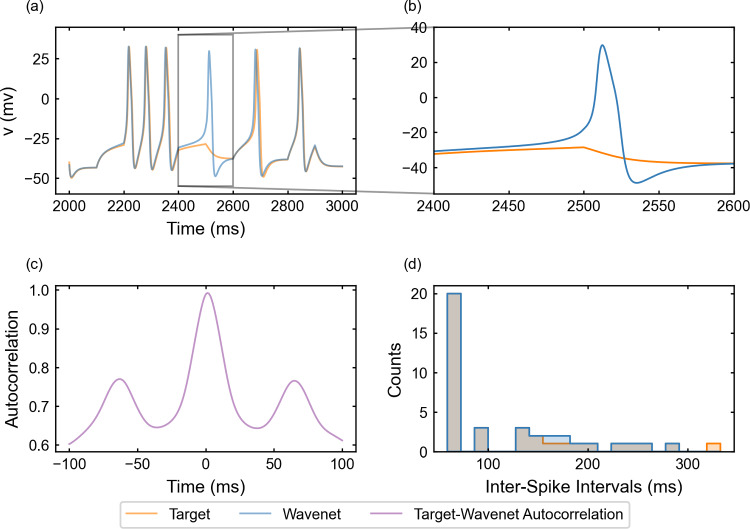
Generalization capability of the wavenet corresponding to the Morris–Lecar model using a stepwise testing input current. Upper panels: Membrane voltage obtained both integrating the model (orange), *v*, and using the wavenet’s prediction (blue), $\widehat{v}$. On the left, comparison in a 1-second window; on the right, a zoom showing the only discrepancy in the detection of spikes. Bottom panels: (left) cross-correlation function between *v* and $\widehat{v}$; (right) comparison of the ISI distributions of *v* (orange) and $\widehat{v}$ (blue), where gray areas indicate full coincidence

### Identification of the neuron models with wavenets and prediction tests: Paradigm I

In this section, we show the main results of the paper within Paradigm I, that is: we identify the neuron models by training the wavenet with the data obtained from integration of the corresponding systems, see “Appendix A”, and using the input current and all variables as input data. In order to generate the data, we use a stepwise $I_{app}$ current trace for training, as the one plotted in Fig. 3. Then, for each model, we use two different types of input testing currents to examine how the wavenet model generalizes, as pointed out in Sect. 2.1.7.

The goodness of the prediction tests is quantified in terms of the different indicators mentioned in Sect. 2.1.6 ($(1 - r^2)$-score, cross-correlation, cosine similarity and ISIs comparison) in order to compare the data predicted by the wavenet with the data generated by the model. In general, these indicators are computed from 5-second simulations withdrawing the first 100 milliseconds in order to avoid transients of the predicted signals. The time windows shown for each model in the figures are adapted in order to highlight the most interesting features.

#### The Morris–Lecar model: training and prediction within Paradigm I

We have identified the Morris–Lecar model by training the wavenet with the data obtained from integration of system (A.1), see “Appendix A.1”. The performance of the identification process for the variable *v* (similar for the other variable, not shown) using a stepwise testing input current is shown in Fig. 6. The $(1 - r^2)$-scores are $(1-r^2)_v \approx 6.3\%$ and $(1-r^2)_w \approx 7.2\%$, whereas the cosine similarity $S_C$ is approximately 0.9903 and 0.9726 for *v* and *w*, respectively. Both indicators confirm an excellent prediction power of the wavenet, as it can be also observed in Fig. 6: most of the time the two trajectories overlap and the discrepancies are insignificant. There is only one notable difference between the two traces a time close to 2500 ms (zoomed in the upper-right panel in Fig. 6), where the wavenet reproduced a spike that was not present in the model simulation. Clearly, this is due to the fact that a small error for voltage values close to the spiking threshold is more critical. However, the subthreshold activity around that spike is captured well. In fact, the cross-correlation function *R* (bottom-left panel in Fig. 6) shows a slight shift to positive values, which can be responsible for the small errors observed. Nevertheless, the high coincidence between the ISI distributions of *v* and $\widehat{v}$ (bottom-right panel in Fig. 6) confirms the high ability of the wavenet to identify the neuron’s dynamics and make accurate predictions.

**Fig. 7:**
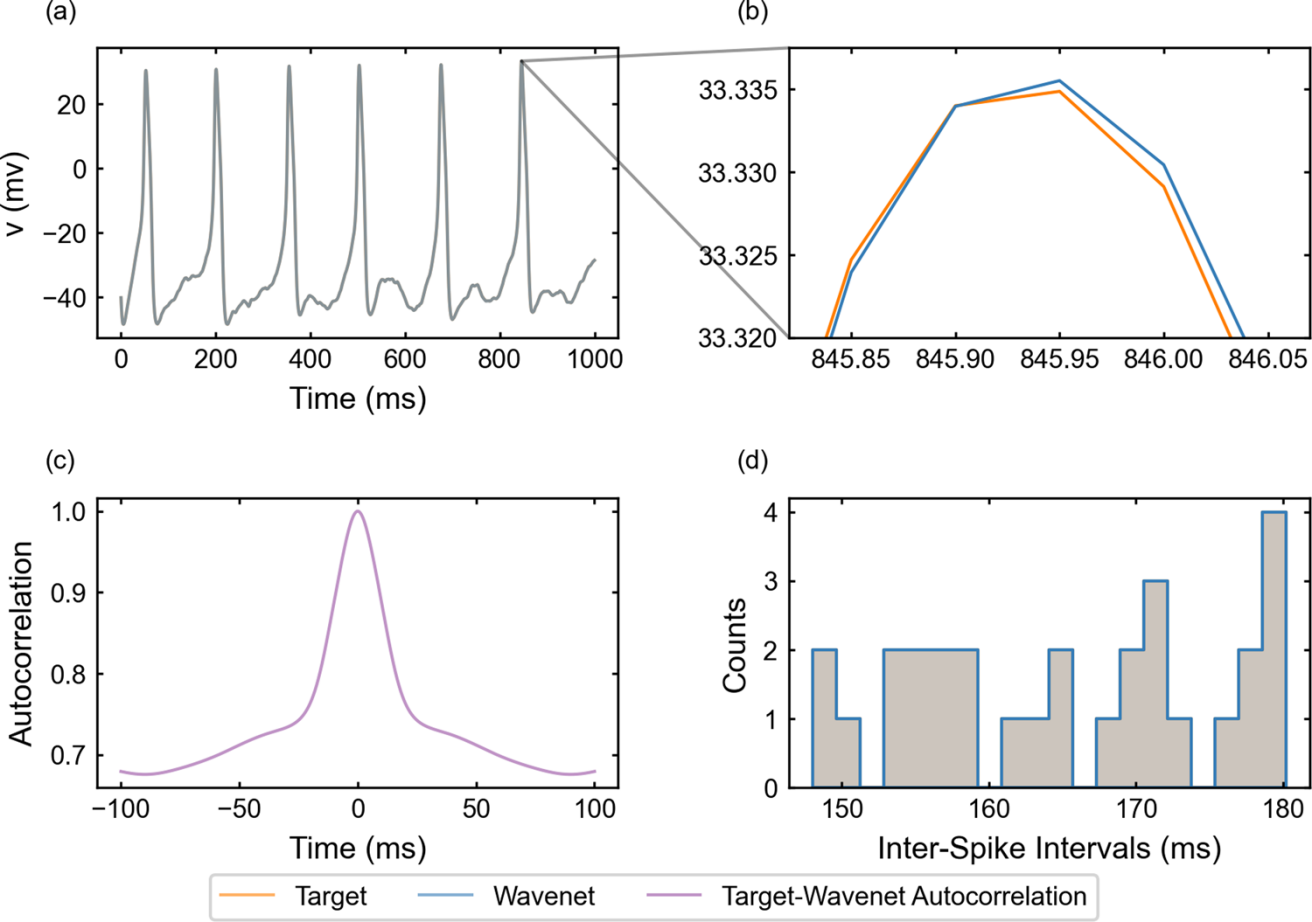
Generalization capability of the wavenet corresponding to the Morris–Lecar model using an oscillatory testing input current. Upper panels: Membrane voltage obtained both integrating the model (orange), *v*, and using the wavenet’s prediction (blue), $\widehat{v}$. On the left, comparison in a 1-second window; on the right, a zoom showing the excellent performance even at the tips of the spikes. Bottom panels: (left) cross-correlation function between *v* and $\widehat{v}$; (right) comparison of the ISI distributions of *v* (orange) and $\widehat{v}$ (blue), where gray areas indicate full coincidence

The oscillatory testing provides even better results. For this purpose, we integrate the system (A.1) again but using a stochastic and oscillating signal as the one defined in (19), see also Fig. 4. Results for the main state variable, *v*, are shown in Fig. 7. In this case, the wavenet prediction is quite more accurate, resulting in scores of $(1-r^2)_v \approx 0.0003\%$ and $(1-r^2)_w \approx 0.0003\%$. whereas the cosine similarity $S_C$ is approximately 1.0000 both for *v* and *w*. We remark that, even though the type of input used for this prediction is qualitatively different from the type of input used to train the wavenet, the performance increases, which confirms the efficiency of the training protocol.

This fact is observed across models as we will show in the next examples. In the upper-left panel of Fig. 7, we barely notice discrepancies between the model and the predicted voltage traces (less than 0.01 mV as shown in the upper-right panel). The cross-correlation function *R* (bottom-left panel) shows an insignificant shift from zero, whereas the ISI distributions of *v* and $\widehat{v}$ (bottom-right panel) exhibit a complete agreement.

#### The FitzHugh–Nagumo model: training and prediction within Paradigm I

**Fig. 8:**
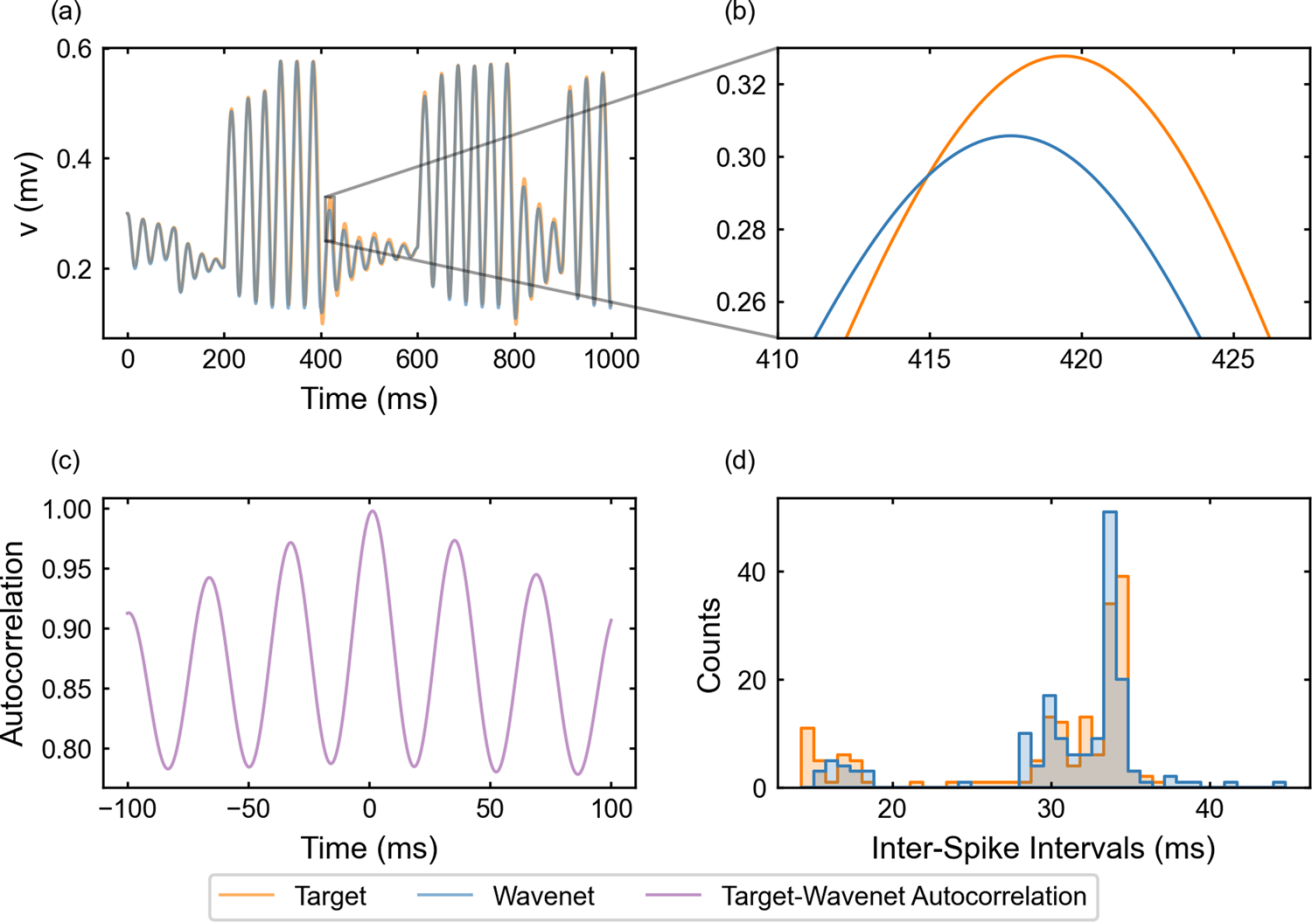
Generalization capability of the wavenet corresponding to the FitzHugh–Nagumo model using a stepwise testing input current. Upper panel: Membrane voltage obtained both integrating the model (orange), *v*, and using the wavenet’s prediction (blue), $\widehat{v}$. On the left, comparison in a 1-second window; on the right, a zoom showing slight discrepancies at the tip of a spike. Bottom panels: (left) cross-correlation function between *v* and $\widehat{v}$; (right) comparison of the ISI distributions of *v* (orange) and $\widehat{v}$ (blue), where gray areas indicate full coincidence

For the identification of the FitzHugh–Nagumo model (see “Appendix A.2”), system (A.4) is integrated following the same procedure as in Sect. 3.2.1. The performance of the identification of the model for the state variable *v* (similar for the other variable, not shown) when using a stepwise testing input current $I_{app}$ is shown in Fig. 8, which contains a 1-second sample of a more complete 5-second simulation (upper-left panel).

The $(1 - r^2)$-scores for the complete simulation are $(1-r^2)_v \approx 6.6\%$ and $(1-r^2)_w \approx 5.7\%$. The only small differences are appreciated at some peaks where the network prediction undershoots and is slightly displaced (see a detail in the upper-right panel of Fig. 8). This feature creates slight differences between the ISI distributions (bottom-right panel in Fig. 8). The cosine similarity $S_C$ is approximately 0.9954 and 0.9970 for *v* and *w*, respectively. Both indicators confirm that the wavenet is able to predict the target trajectories. Due to the relatively fast (around 30Hz) and regular oscillations of the neuron’s response, the cross-correlation function *R* (bottom-left panel) shows several peaks at multiples of the approximated period.

**Fig. 9:**
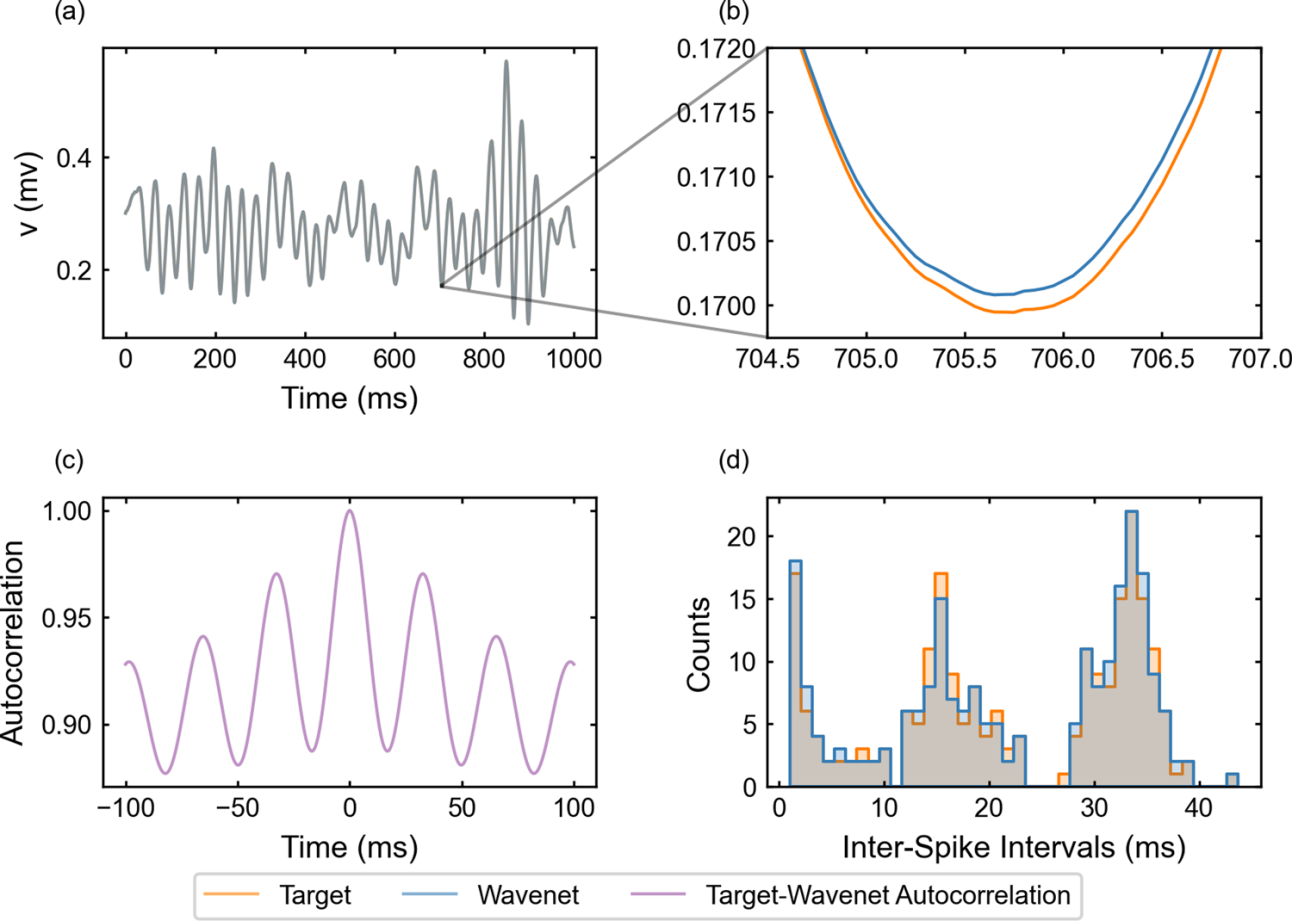
Generalization capability of the wavenet corresponding to the FitzHugh–Nagumo model using an oscillatory testing input current. Upper panel: Membrane voltage obtained both integrating the model (orange), *v*, and using the wavenet’s prediction (blue), $\widehat{v}$. On the left, comparison in a 1-second window; on the right, a zoom showing the largest inaccuracy found. Bottom panels: (left) cross-correlation function between *v* and $\widehat{v}$; (right) comparison of the ISI distributions of *v* (orange) and $\widehat{v}$ (blue), where gray areas indicated full coincidence

We now examine the generalization of the network under the oscillatory testing input current. The resulting plots for the state variable *v* (similar for *w*, not shown) are displayed in Fig. 9. Again, one can see that the wavenet is able to predict the behavior of the model dynamics (see upper-left panel), in this case being able to even capture the target dynamics at the peaks and troughs (see upper-right panel). The prediction for both variables *v* and *w* produced the $(1 - r^2)$-scores $(1-r^2)_v \approx 0.0025\%$ and $(1-r^2)_w \approx 0.0023\%$, respectively. The cosine similarity $S_C$ is approximately 1.000 both for *v* and *w* and the cross-correlation function *R* is sensitive to the relative high-frequency oscillations (bottom-left panel). The ISI distributions (bottom-right panel) show also a high agreement.

We observe that there is a noticeable difference between the predictions of the dynamics resulting from the stepwise (see Figs. 6 and 8) and the stochastic signals (see Figs. 7 and 9). The stochastic input predictions are more accurate (lower $(1 - r^2)$-score), probably due to the smooth oscillatory basis used to generate the stochastic input, which makes its variation less abrupt, whereas the design of the stepwise input allows sudden excursions from lower to the higher values within the admissible range of currents. This phenomenon also occurs in the following (3D) examples, consistently showing the difficulty of the wavenet to identify abrupt changes in the dynamics.

#### The FitzHugh–Nagumo–Rinzel model: training and prediction within Paradigm I

**Fig. 10:**
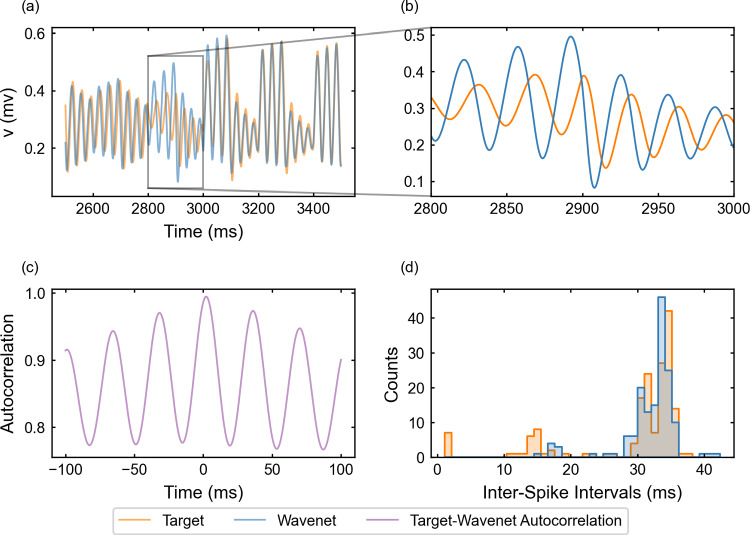
Generalization capability of the wavenet corresponding to the FitzHugh–Nagumo–Rinzel model using a stepwise testing input current. Upper panel: Membrane voltage obtained both integrating the model (orange), *v*, and using the wavenet’s prediction (blue), $\widehat{v}$. On the left, comparison in a 1-second window; on the right, a zoom showing the most critical discrepancies. Bottom panels: (left) cross-correlation function between *v* and $\widehat{v}$; (right) comparison of the ISI distributions of *v* (blue) and $\widehat{v}$ (orange), where gray areas indicated full coincidence

Following the same procedure as in the examples above, we now study the FitzHugh–Nagumo 3D model (A.5), see “Appendix A.3”. Notice that we are now handling a three-dimensional system with an additional (slow) timescale through the variable *y*, which makes transient activity between different $I_{app}$ values more relevant. From a computational point of view, this turns into a harder *learning* process and so, it has implied an increment of integration steps, which means significantly more data. The identification results for the variable *v* (similar for the other variables, not shown) are displayed in Fig. 10. We can appreciate that the increase in dimensionality and the presence of a new timescale compromise the goodness of the prediction. Nonetheless, the wavenet is able to qualitatively follow the target dynamics. The identification scores for this model are $(1-r^2)_v \approx 16.01\%$, $(1-r^2)_w \approx 14.15\%$ and $(1-r^2)_y \approx 2.203\%$. In the upper-right panel of Fig. 10, we display how, in some time regions, the predicted trace (blue) overshoots and has a noticeable displacement to the left; however, the ISI distributions (bottom-right panel) still exhibit a high level of coincidence. The cosine similarity $S_C$ is approximately 0.9885, 0.9923 and 1.0000 for *v*, *w* and *y*, respectively. Similar to the FHN2D model studied above, the cross-correlation function *R* (bottom-left panel) shows again different peaks related to the relatively high-frequency oscillations, but the peak at 0-lag is still prominent.

**Fig. 11:**
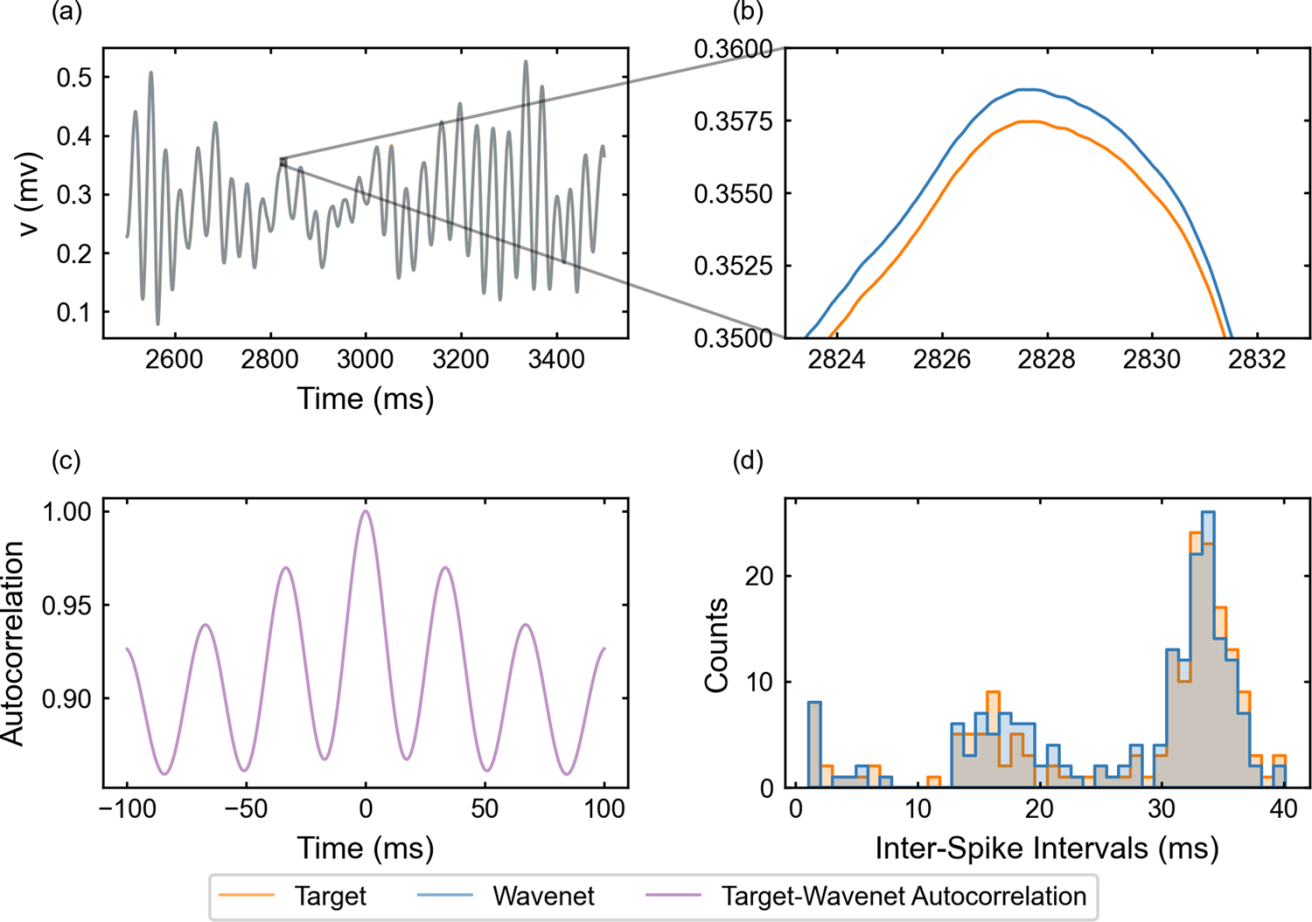
Generalization capability of the wavenet corresponding to the FitzHugh–Nagumo–Rinzel model using an oscillatory testing input current. Upper panel: Membrane voltage obtained both integrating the model (orange), *v*, and using the wavenet’s prediction (blue), $\widehat{v}$. On the left, comparison in a 1-second window; on the right, a zoom showing maximal discrepancies, which are insignificant. Bottom panels: (left) cross-correlation function between *v* and $\widehat{v}$; (right) comparison of the ISI distributions of *v* (blue) and $\widehat{v}$ (orange), where gray areas indicated full coincidence

The results of the generalization of the network under the oscillatory testing input current are shown in Fig. 11. As with the 2D FitHugh–Nagumo model, we observe a much better approximation, with $(1 - r^2)$-scores $(1-r^2)_v \approx 0.0098\%$, $(1-r^2)_w \approx 0.0093\%$ and $(1-r^2)_y \approx 0.0086\%$. The cosine similarity $S_C$ is approximately 1.000 for all variables, *v*, *w* and *y*. The cross-correlation function *R* (bottom-left panel) shows the usual peaks related to the relatively high-frequency oscillations and the ISI distributions (bottom-right panel) exhibit again a strong overlap.

**Fig. 12:**
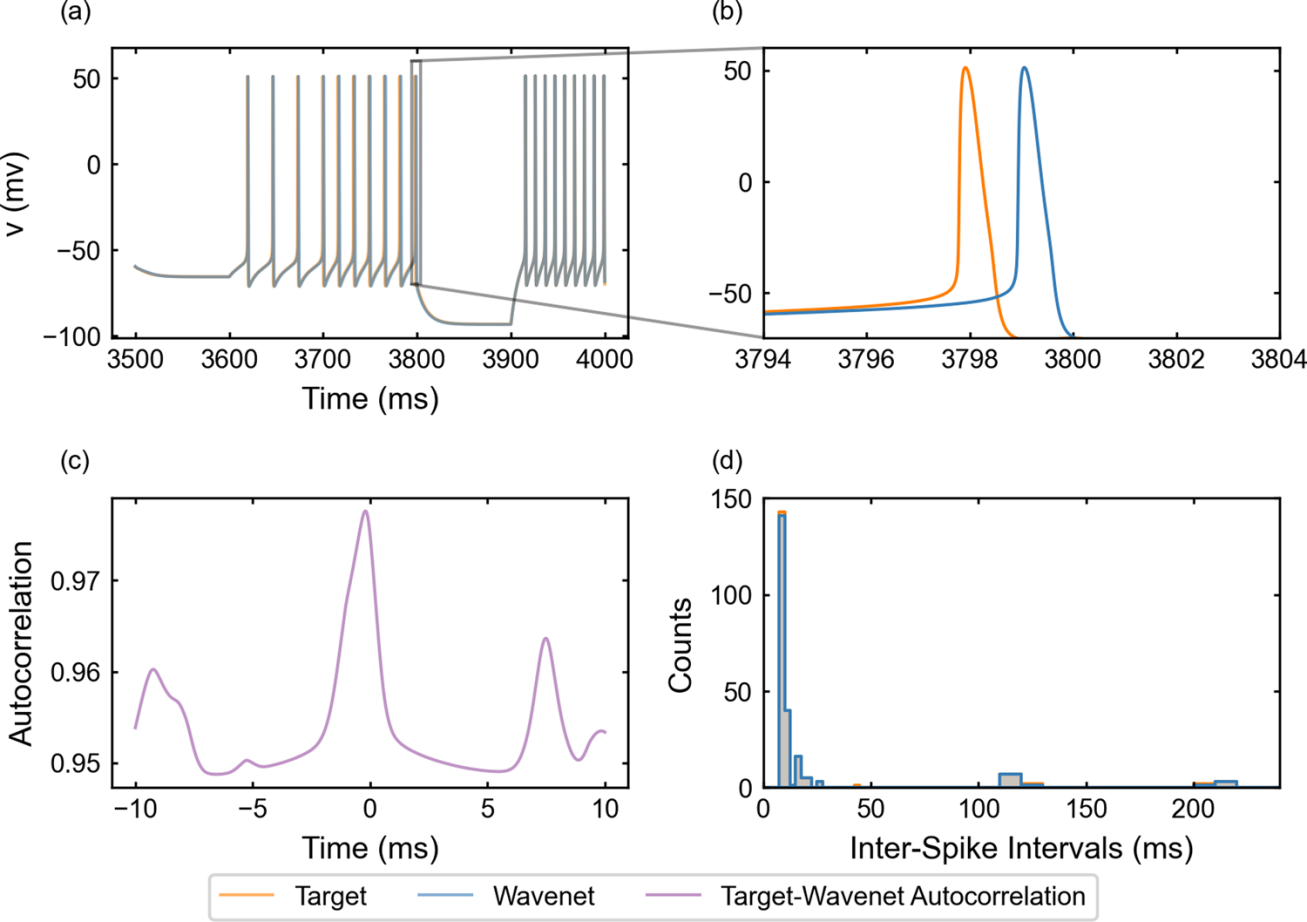
Generalization capability of the wavenet corresponding to the Wang model using a stepwise testing input current. Upper panel: Membrane voltage obtained both integrating the model (orange), *v*, and using the wavenet’s prediction (blue), $\widehat{v}$. On the left, comparison in a 500-milliseconds window; on the right, a zoom showing a small delay in the spike-time prediction. Bottom panels: (left) cross-correlation function between *v* and $\widehat{v}$; (right) comparison of the ISI distributions of *v* (blue) and $\widehat{v}$ (orange), where gray areas indicated full coincidence

#### The Wang model: training and prediction within Paradigm I

Finally, for the wavenet identification of the Wang model (see “Appendix A.4”), we use Eqs. (A.6) and (A.7) with parameter values from (A.8) and (A.9). The results of the generalization for the variable *v* (similar for the other variables, not shown) under the stepwise testing input current are shown in Fig. 12. It is easily verified that the wavenet is able to mimic the target dynamics qualitatively most of the time. However, we observe some small (about 1 ms) delays in the spike-time prediction (see upper-right panel). As with the FitzHugh–Nagumo–Rinzel model, the increase of dimensionality implies important computational demands, which turn as well into relatively large $(1 - r^2)$-scores, $(1-r^2)_v \approx 65.06\%$, $(1-r^2)_h \approx 60.69\%$ and $(1-r^2)_n \approx 56.29\%$, obtained along a 5-second simulation. The persistent delay in the spike-time prediction may be the cause of these bad scores. Looking at the other indicators, we can assess the good quality of the identification. On the one side, the cosine similarity $S_C$ is 0.9750, 0.9895 and 0.7874 for *v*, *h* and *n*, respectively. Note that we get excellent agreements for the voltage *v* and the sodium gating variable *h*. However, for the potassium gating variable, *n*, the cosine similarity decreases significantly; the fact that *n* is specially active during the hyperpolarization regime is probably increasing its sensitivity to the spike-time delays. On the other side, the cross-correlation function *R* (bottom-left panel) also shows a prominent peak at zero and the ISI distributions (bottom-right panel) exhibit again a strong overlap, which confirms that, even though some indicators raise a warning message, the wavenet essentially captures the neuron dynamics. It is worth noticing that the cross-correlation function is less symmetric than in other cases, showing secondary peaks close to $-9$ ms and $+8$ ms; the presence of the peaks is due to relatively high-frequency spiking activity while the asymmetry is probably a side effect of a slight variability of the abovementioned spike-time delays.

**Fig. 13:**
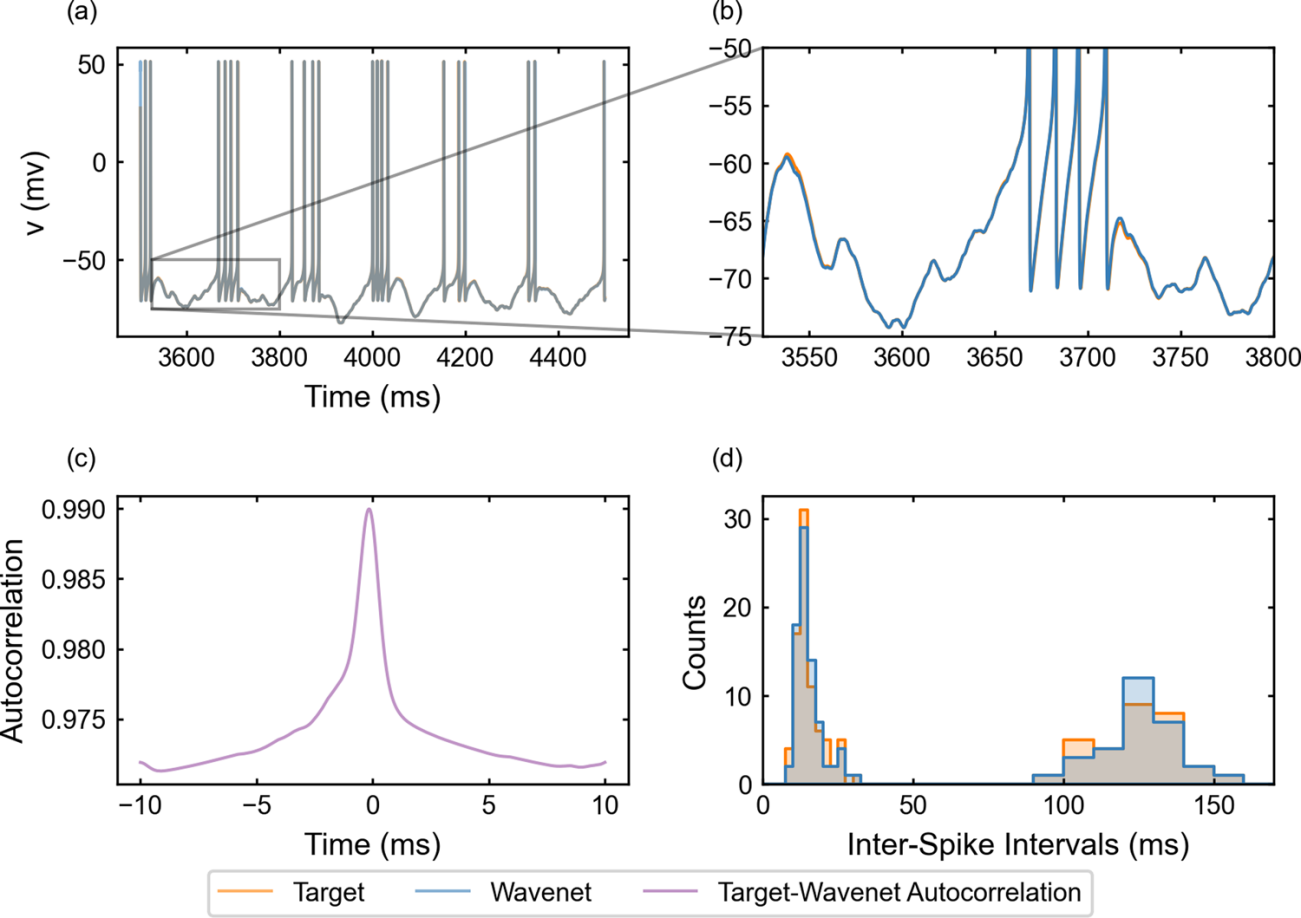
Generalization capability of the wavenet corresponding to the Wang model using an oscillatory testing input current. Upper panel: Membrane voltage obtained both integrating the model (green) and using the wavenet’s prediction (orange). On the left, comparison in a 1-second window; on the right, a zoom showing the accumulated delay in the prediction of *v*. Lower panels: Same plot for the gating variables *h* (left panel) and *n* (right panel) along 1 second. The $(1 - r^2)$-scores are computed for the complete 5-second simulation

We train the network now under the oscillatory testing input current. The resulting plots for the state variables *v* (similar for *h* and *n*, not shown) are displayed in Fig. 13. The results improve, as in the previous example, those obtained with the stepwise testing input current. The $(1 - r^2)$-scores are still high, $(1-r^2)_v \approx 59.43\%$, $(1-r^2)_h \approx 44.39\%$ and $(1-r^2)_n \approx 43.57\%$, but we observe a better agreement between the trajectory obtained from the model and the prediction provided by the wavenet (see upper panels of Fig. 13). The cosine similarity $S_C$ is 0.9891, 0.9961 and 0.8398 for *v*, *h* and *n*, respectively. Note that the cosine similarity for *n* has increased with respect to the stepwise testing input current but it is still low. The cross-correlation function *R* (bottom-left panel) shows a nice sharp peak at zero, whereas the ISI distributions (bottom-right panel) exhibit again a strong overlap, which confirms again the good performance of the wavenet.

### Identification of the neuron models with wavenets and prediction tests: Paradigm II

In this section, we show the main results of the paper within Paradigm II, that is: we identify the neuron models by training the wavenet with the data obtained from integration of the corresponding systems, see “Appendix A”, and using only the input current and the voltage variable as input data (see the scheme in Fig. 1c). The testing procedures (stepwise and oscillatory) and all the indicators ($(1 - r^2)$-score and cosine similarity) are the same as in Sect. 3.2. Obviously, in this paradigm we only obtain results for the voltage variable. At this stage, we can present satisfactory results for the 2D models (Morris–Lecar and Fitzhugh–Nagumo); we show anyway the results for the Fitzhugh–Nagumo–Rinzel model to illustrate the problems that arise for higher-dimensional data. For the 2D models, the input data consisted of input current plus the current voltage value and the preceding one ($q=1$ in the notation established in Sect. 2.1.1). For the Fitzhugh–Nagumo–Rinzel model, we have taken $q=4$, that is, we have used $v_j$, for $j=k-3,k-2,k-1,k$, in order to predict the next state ($v_{k+1}$). We note that the resulting wavenet contains 7780 wavelons, with one resolution level and only 3 overlapping functions. If we were to use 4 overlapping functions, the network would increase up to 32,772 wavelons; if we maintained the number of overlapping functions but increased the resolution, then the network would scale up to 66586 wavelons. We have preferred to show the results obtained at a lower computational cost.

#### The Morris–Lecar model: training and prediction within Paradigm II

**Fig. 14:**
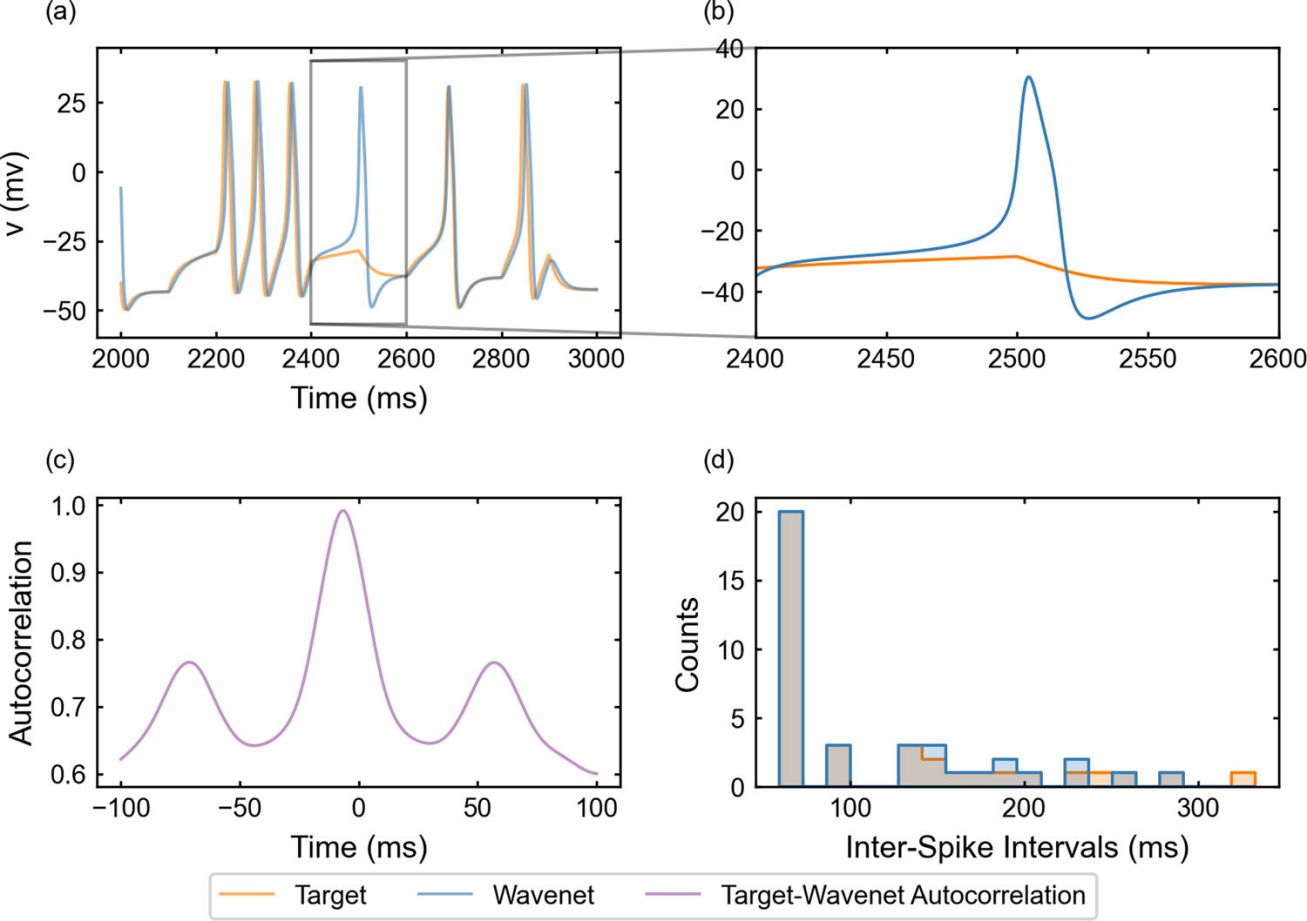
Generalization capability of the wavenet corresponding to the Morris–Lecar model using only the external current and the voltage as inputs and the stepwise testing input. Upper panels: Membrane voltage obtained both integrating the model (orange), *v*, and using the wavenet’s prediction (blue), $\widehat{v}$. On the left, comparison in a 1-second window; on the right, a zoom showing the only discrepancy in the detection of spikes. Bottom panels: (left) cross-correlation function between *v* and $\widehat{v}$; (right) comparison of the ISI distributions of *v* (orange) and $\widehat{v}$ (blue), where gray areas indicate full coincidence

The performance of the identification process for the variable *v* using a stepwise testing input current is shown in Fig. 14. The $(1 - r^2)$-score is approximately $52.22\%$, whereas the cosine similarity $S_C$ is approximately 0.9196.

We observe that $(1 - r^2)$-score decays considerably with respect to the corresponding value for Paradigm I, but all other indicators show a high performance: the cosine similarity keeps at high values and the ISI distributions are similar (compare Figs. 6c and 14c). The worsening of the $(1 - r^2)$-score may be due to small delays in the prediction. We must take into account that using previous values of the voltage instead of the values of the auxiliary variables may induce a delay; this is confirmed by the autocorrelation function shown in Fig. 14c, which is clearly not centered at zero. As in Paradigm I, we note that the wavenet generates a spike that was not present in the model simulation.

**Fig. 15:**
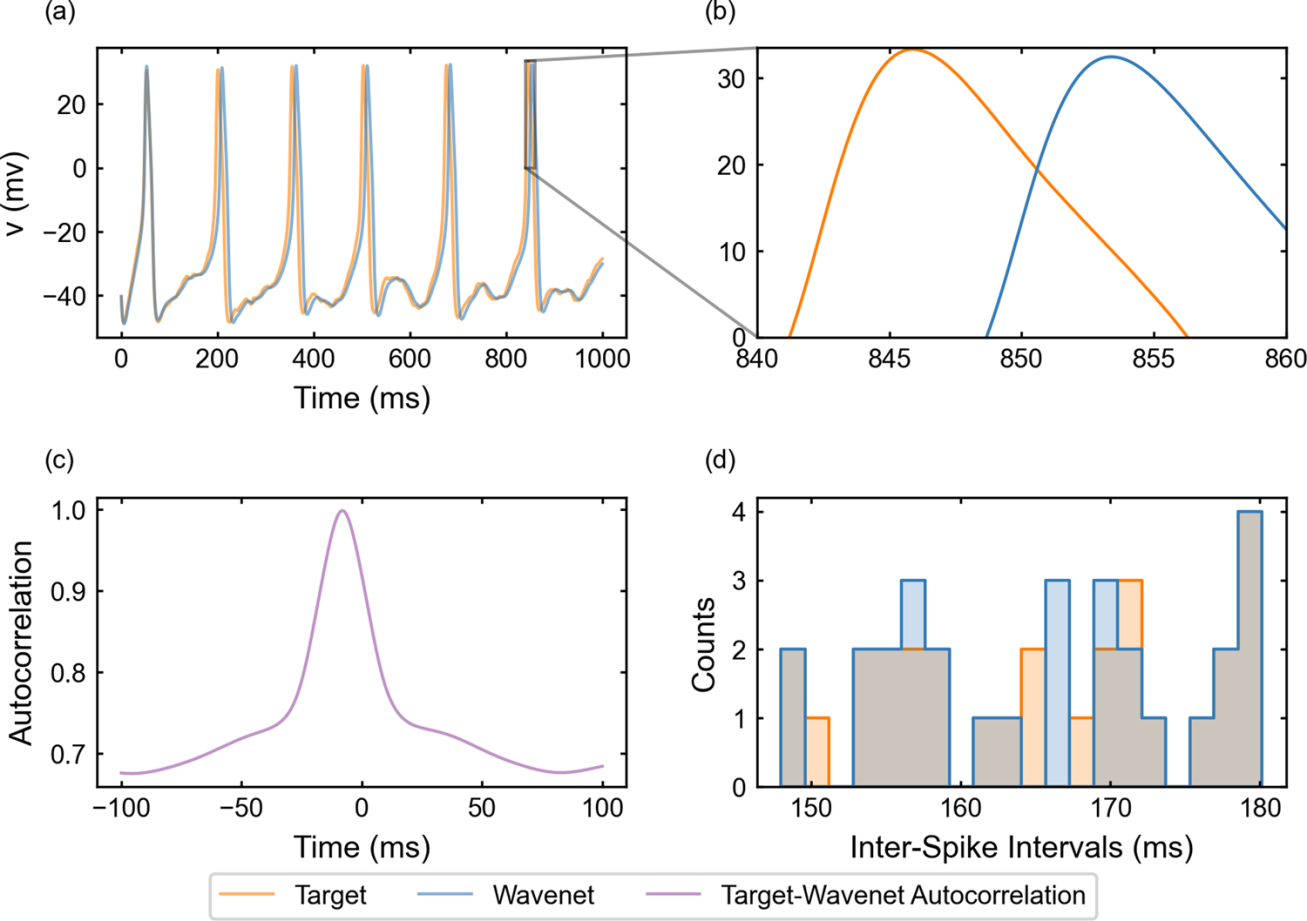
Generalization capability of the wavenet corresponding to the Morris–Lecar model using only the external current and the voltage as inputs and the oscillatory testing input. Upper panels: Membrane voltage obtained both integrating the model (orange), *v*, and using the wavenet’s prediction (blue), $\widehat{v}$. On the left, comparison in a 1-second window; on the right, a zoom showing an instance of the slight delay of the prediction. Bottom panels: (left) cross-correlation function between *v* and $\widehat{v}$; (right) comparison of the ISI distributions of *v* (orange) and $\widehat{v}$ (blue), where gray areas indicate full coincidence

The oscillatory testing provides similar results, see Fig. 15. In panel (a), we observe again a clear matching in agreement with the value of the cosine similarity, $S_C\approx 0.9210$; however, it is not reflected in the $(1 - r^2)$-score, $(1-r^2) \approx 65.51\%$. The explanations provided for the stepwise testing are applicable also here. Compared to Paradigm I, we observe a lower performance of the ISI prediction. This may be due to lags in the prediction (again, the autocorrelation is clearly centered away from zero, see Fig. 15c), which have slightly different lengths over time and shift the prediction to a neighboring bin of the histogram.

#### The FitzHugh–Nagumo model: training and prediction within Paradigm II

**Fig. 16:**
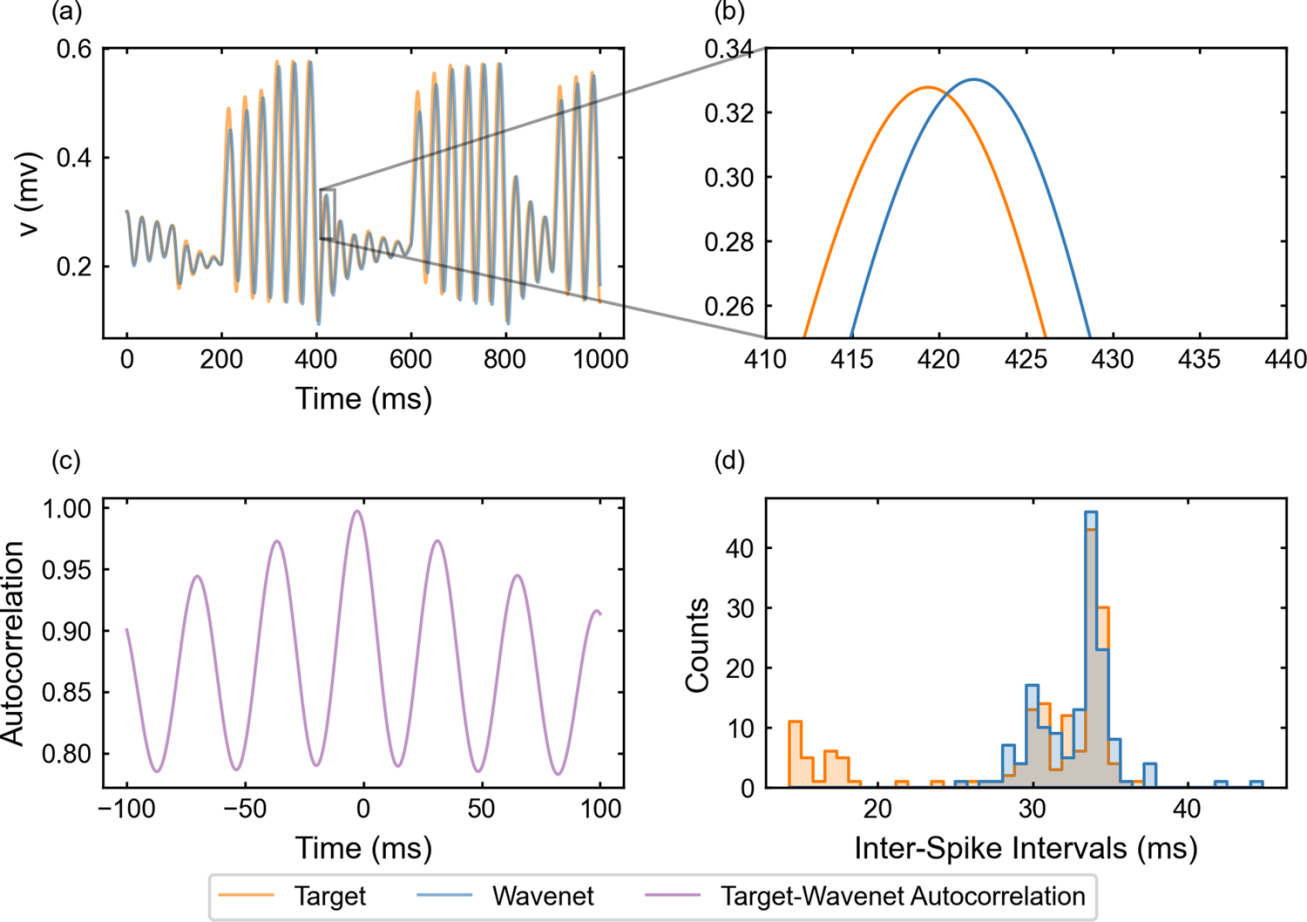
Generalization capability of the wavenet corresponding to the Fitzhugh–Nagumo model using only the external current and the voltage as inputs and the stepwise testing input. Upper panels: Membrane voltage obtained both integrating the model (orange), *v*, and using the wavenet’s prediction (blue), $\widehat{v}$. On the left, comparison in a 1-second window; on the right, a zoom showing the small delay in the wavenet prediction. Bottom panels: (left) cross-correlation function between *v* and $\widehat{v}$; (right) comparison of the ISI distributions of *v* (orange) and $\widehat{v}$ (blue), where gray areas indicate full coincidence

The performance of the identification process for the variable *v* using a stepwise testing input current is shown in Fig. 16. The $(1 - r^2)$-score is approximately $24.38\%$, whereas the cosine similarity $S_C$ is approximately 0.9828. The results are slightly better than for the Morris–Lecar model studied in the last section. It is worth mentioning that, compared to Paradigm I (see Fig. 8d), the ISI distributions do not match for the shortest ISIs but show a satisfactory match for intervals larger than 20 units of time, which are the most common ones.

**Fig. 17:**
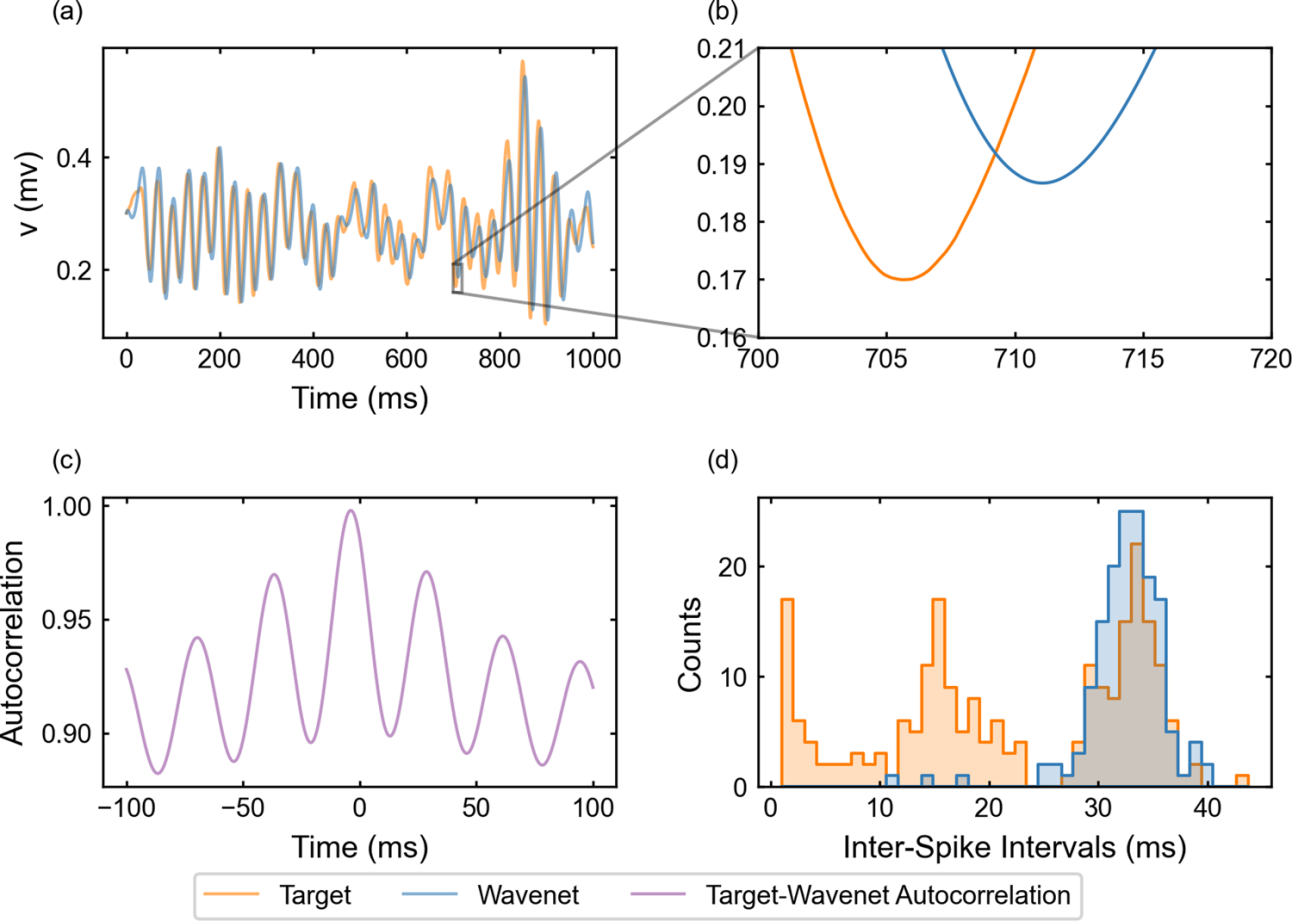
Generalization capability of the wavenet corresponding to the Fitzhugh–Nagumo model using only the external current and the voltage as inputs and the oscillatory testing input. Upper panels: Membrane voltage obtained both integrating the model (orange), *v*, and using the wavenet’s prediction (blue), $\widehat{v}$. On the left, comparison in a 1-second window; on the right, a zoom showing typical inaccuracies in the wavenet prediction. Bottom panels: (left) cross-correlation function between *v* and $\widehat{v}$; (right) comparison of the ISI distributions of *v* (orange) and $\widehat{v}$ (blue), where gray areas indicate full coincidence

For the oscillatory testing, see Fig. 17, the $(1 - r^2)$-score is approximately $44.28\%$, whereas the cosine similarity $S_C$ is approximately 0.9853. Panel (d) confirms that lower ISIs are not captured by the network; in contrast to the stepwise testing, lower ISIs are more numerous because, by design, the oscillatory input elicits more activity with short amplitude and higher frequency in this model.

#### The FitzHugh–Nagumo–Rinzel model: training and prediction within Paradigm II

**Fig. 18:**
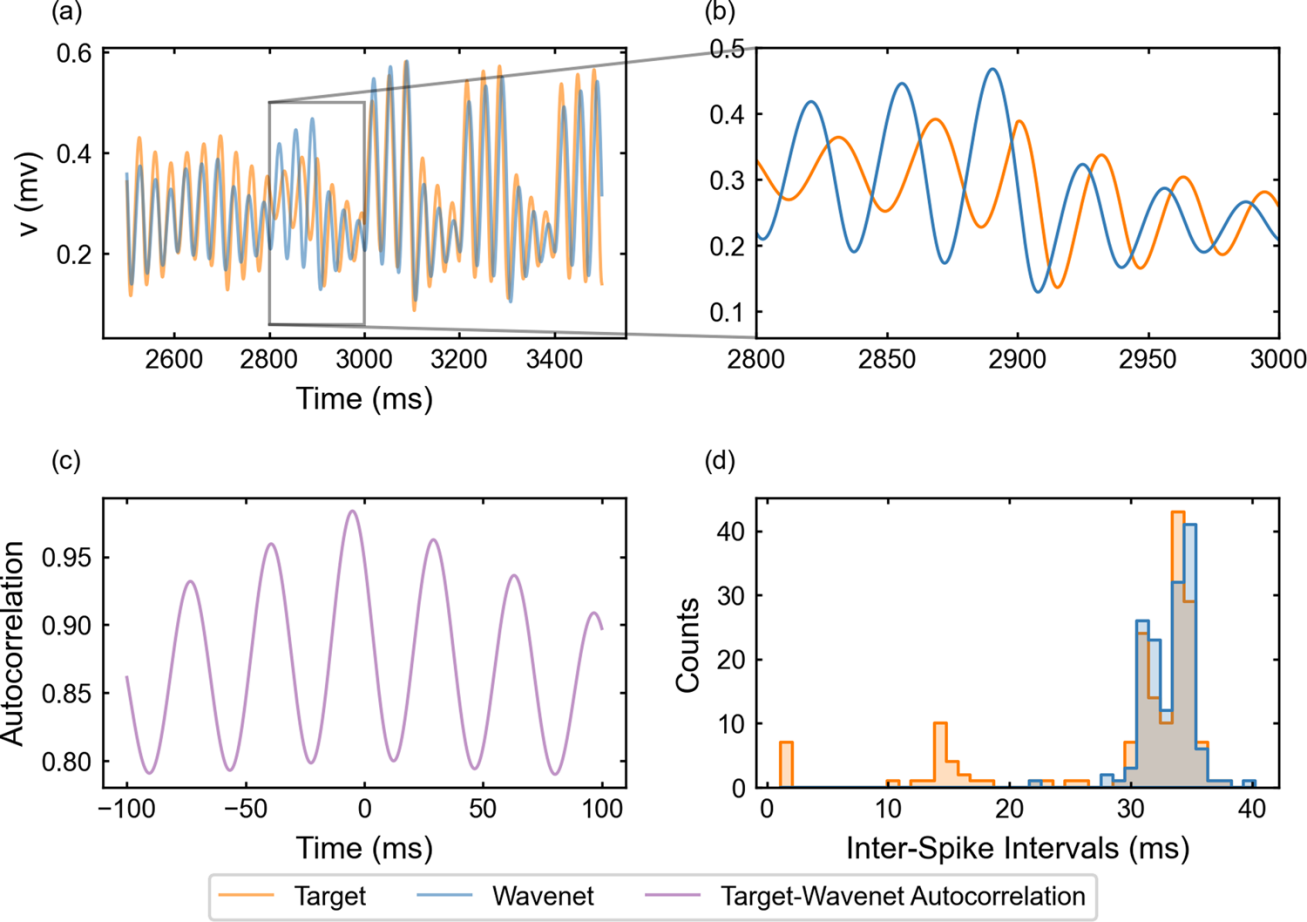
Generalization capability of the wavenet corresponding to the FitzHugh–Nagumo–Rinzel model using only the external current and the voltage as inputs and the stepwise testing input. Upper panel: Membrane voltage obtained both integrating the model (orange), *v*, and using the wavenet’s prediction (blue), $\widehat{v}$. On the left, comparison in a 1-second window; on the right, a zoom showing maximal discrepancies, which combine phase-lag and small inaccuracies in amplitude. Bottom panels: (left) cross-correlation function between *v* and $\widehat{v}$; (right) comparison of the ISI distributions of *v* (blue) and $\widehat{v}$ (orange), where gray areas indicated full coincidence

The performance of the identification process for the variable *v* using a stepwise testing input current is shown in Fig. 18. The $(1 - r^2)$-score is approximately $75.58\%$, whereas the cosine similarity $S_C$ is approximately 0.9449. The prediction signal is similar to the target and the identification fails in the same $I_{app}$ regions as in Fig. 10. In some regions, we observe a combination of phase-lag and small inaccuracies in amplitude. As for the preceding model in Sect. 3.3.2, the lower ISIs are not captured by the network. The most noticeable difference with the 2D examples and also with the same model for Paradigm I is the autocorrelation function which has lost the harmonics.

**Fig. 19:**
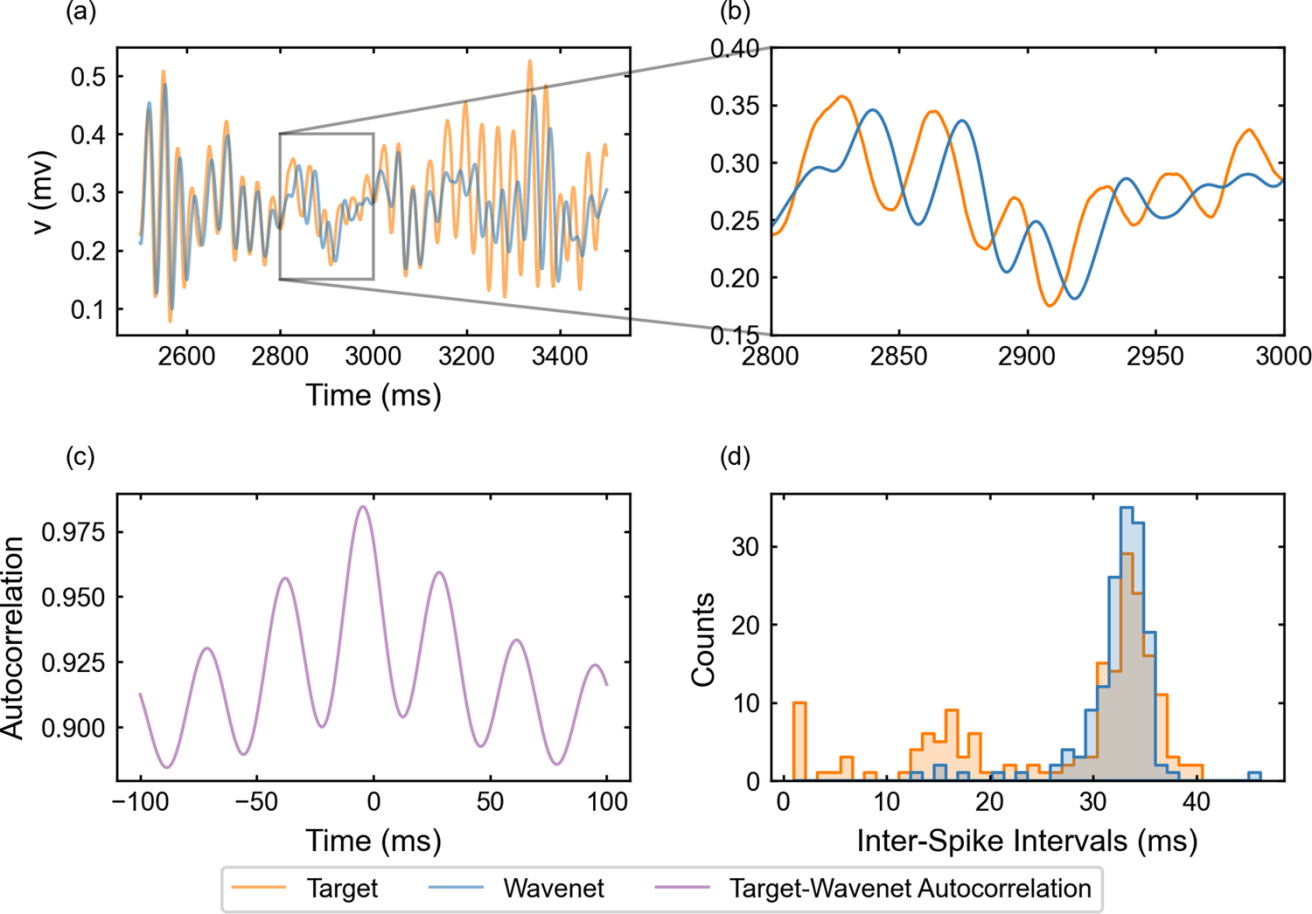
Generalization capability of the wavenet corresponding to the FitzHugh–Nagumo–Rinzel model using only the external current and the voltage as inputs and the oscillatory testing input. Upper panel: Membrane voltage obtained both integrating the model (orange), *v*, and using the wavenet’s prediction (blue), $\widehat{v}$. On the left, comparison in a 1-second window; on the right, a zoom showing the onset of relevant discrepancies. Bottom panels: (left) cross-correlation function between *v* and $\widehat{v}$; (right) comparison of the ISI distributions of *v* (blue) and $\widehat{v}$ (orange), where gray areas indicated full coincidence

**Fig. 20:**
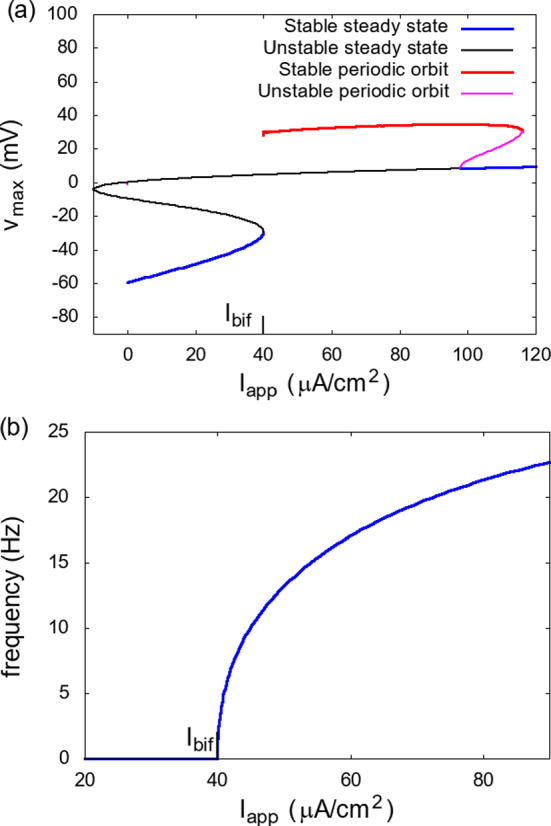
Bifurcation diagram of system (A.1) in terms of the parameter $I_{app}$. All other parameter values are given in (A.3). **a** The graph shows the maximal value of the variable *v* on the equilibrium points or on the periodic orbits which, indeed, are limit cycles. **b** Frequency of oscillation

**Fig. 21:**
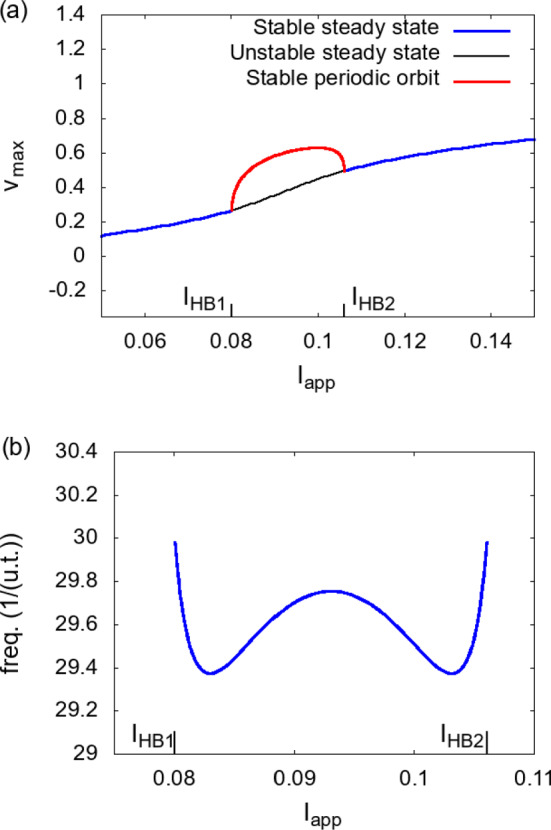
Bifurcation diagram of system (A.4) in terms of the parameter $I_{app}$. **a** The graph shows the maximal value of the variable *v* on the equilibrium points or on the periodic orbits which, indeed, are limit cycles. **b** Frequency of oscillation. The system presents two Hopf bifurcations at $I_{HB1}\approx 0.08008$ and $I_{HB2}\approx 0.106044$

**Fig. 22:**
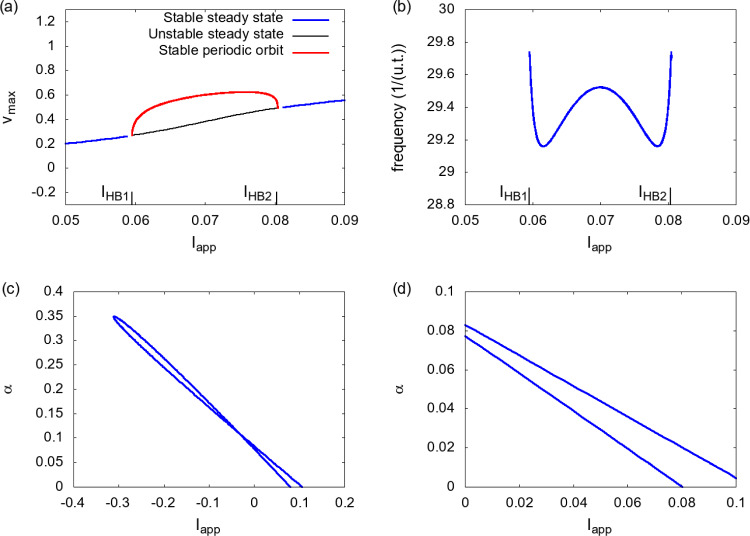
Bifurcation diagrams of system (A.5). Upper row: in terms of the parameter $I_{app}$. **a** The graph shows the maximal value of the variable *v* on the equilibrium points or on the periodic orbits which, indeed, are limit cycles. **b** Frequency of oscillation. The system presents two Hopf bifurcations at $I_{HB1}\approx 0.0595108$ and $I_{HB2}\approx 0.0803052$. Lower row: in terms of parameters $I_{app}$ and $\alpha$. **c** Full-scale visualization to show the two Hopf bifurcation curves on the two-parameter bifurcation diagram. **d** Visualization restricted to $I_{app}\ge 0$ and $\alpha \ge 0$

**Fig. 23:**
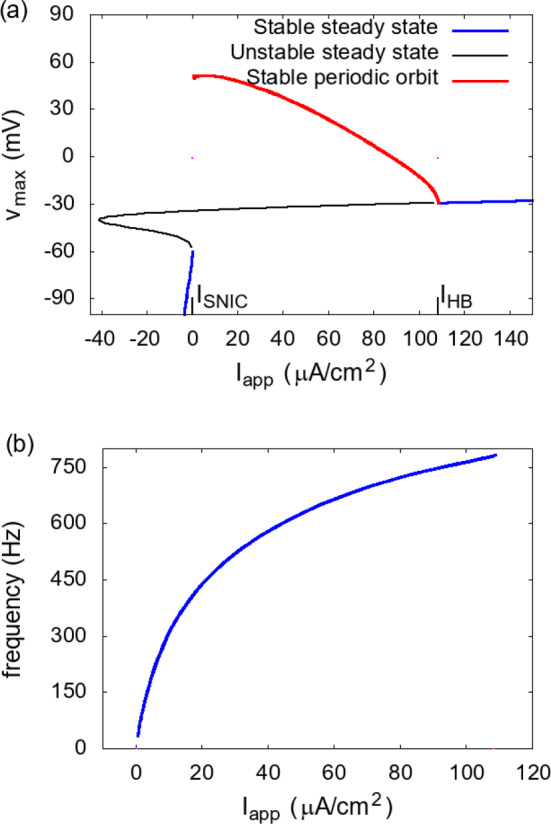
Bifurcation diagram of system (A.6) in terms of the parameter $I_{app}$. **a** The graph shows the maximal value of the variable *v* on the equilibrium points or on the periodic orbits which, indeed, are limit cycles. **b** Frequency of oscillation. The system presents a SNIC bifurcation at $I_{app}\approx 0.225653$ and a Hopf bifurcation at $I_{app}\approx 108.2843$

For the oscillatory testing, see Fig. 19, the $(1 - r^2)$-score is approximately $62.64\%$, whereas the cosine similarity is $S_C\approx 0.9753$. Here, the phase-lag and inaccuracies shown in Fig. 19b are more persistent along the simulation.

## Conclusion

**Table 4:** Summary of the comparisons between target and predicted variables using the $(1 - r^2)$-score and the cosine similarity indicators

Model	$(1 - r^2)$-score (%)	Cosine similarity
ML_step_	(6.3, 7.2)	(0.990, 0.973)
ML_osc_	(0.0003, 0.0003)	(1.00, 1.00)
FHN_step_	(6.6, 5.7)	(0.995, 0.997)
FHN_osc_	(0.0025, 0.0023)	(1.00, 1.00)
FHN3D_step_	(16.01, 14.15, 2.20)	(0.989, 0.992, 1.00)
FHN3D_osc_	(0.0098, 0.0093, 0.0086)	(1.00, 1.00, 1.00)
Wang_step_	(65.06, 60.69, 56.29)	(0.975, 0.990, 0.787)
Wang_osc_	(59.43, 43.39, 43.57)	(0.989, 0.996, 0.840)

For each model, we give the results for the two different stimuli type: stepwise (*step*) and oscillatory (*osc*). The components of each vector refer to the 2 or 3 variables of the model, that is, (*v*, *w*) for the Morris–Lecar (ML) model and for the Fitzhugh–Nagumo (FHN) model, (*v*, *w*, *y*) for the Fitzhugh–Nagumo–Rinzel (FHN3D) model and (*v*, *h*, *n*) for the Wang model. All values are approximate

We have applied an ANN based on wavelets (called *wavenet*) to identify the dynamics of several neuron models: Morris–Lecar, the FitzHugh–Nagumo, a three-dimensional version of FitzHugh–Nagumo and a model of a pyramidal neuron in two different paradigms (I and II) according to whether we consider all the state variables for the training or only the voltage one. The weights of the network were obtained by solving a least-square optimization problem, which is linear in the parameters, thus ensuring a global solution. The datasets used for the optimization procedure were obtained from simulations of the neuron models under a stepwise stimulus that swept the biological plausible interval of the applied current. To assess the performance of the network, we simulated new trajectories using two different stimuli types, one similar to the *training* protocol and another one generated by means of an oscillatory stochastic differential equation. Then, the solutions obtained from the neuron models (target) were compared with the solutions provided by the *trained* wavenet (prediction) using different indicators (a regression score, the cosine similarity and ISI distribution); cross-correlation functions of the two traces were also monitored. The values of these indicators for all models analyzed are summarized in Table 4 for Paradigm I and Table 5 for Paradigm II. Regarding Paradigm I, for the 2D models (Morris–Lecar and Fitzhugh–Nagumo) even the regression score, the most sensitive indicator to small errors in the predictions gives excellent results. By analyzing the 3D models (Fitzhugh–Nagumo–Rinzel and Wang), we detect two factors that make predictions more difficult: the increase of dimensionality and the timescales separation. For both models, the regression score worsens significantly compared to the 2D models, but specially for the Wang model. Recall that the Wang model is more realistic (pyramidal cell model) and presents sharper spikes (relaxation-oscillation-like) than the Fitzhugh–Nagumo–Rinzel model; this is possibly the reason of the loss of accuracy and indicates the influence of timescales in the identification of the neuronal dynamics since the discrepancies found in the comparison between target and predicted trajectories concentrate around the spike times. However, even for these two 3D models, the other indicators (cosine similarity and ISI distribution comparison) still confirm a high performance of our wavenet in capturing the neuron dynamics. Paradigm II is more challenging since we avoid using the non-measurable variables which requires to extract more information from the voltage trace. For this purpose, we use previous iterates of the voltage. For the Morris–Lecar and Fitzhugh–Nagumo models, one previous iterate is enough to obtain excellent results: compared to Paradigm I, only the most sensitive score, $(1 - r^2)$-score, gets worse. For the Fitzhugh–Nagumo model, the lowest ISIs (very few of them) are not well captured by the wavenet. For the 3D cases, having an additional (slow) time scale makes the identification more difficult. We still do not obtain completely satisfactory results, but we show those obtained with the Fitzhugh–Nagumo–Rinzel model using four previous voltage values as input data: the cosine similarity is still acceptable, the ISI distribution shows satisfactory agreement for intervals larger than 20 time units, which are the most common, but the autocorrelation function loses the harmonics.

**Table 5:** Summary of the comparisons between target and predicted variables using the $(1 - r^2)$-score and the cosine similarity indicators in Paradigm II

Model	$(1 - r^2)$-score (%)	**Cosine similarity**
ML_step_	52.22	0.9196
ML_osc_	65.51	0.9210
FHN_step_	24.38	0.9828
FHN_osc_	44.28	0.9853
FHN3D_step_	75.58	0.9449
FHN3D_osc_	62.64	0.9753

For each model, we give the results for the two different stimuli type: stepwise (*step*) and oscillatory (*osc*). All indicators refer to the variable *v*

## Discussion

We have explored the potentiality of a specific family of artificial neural networks to identify and predict neuronal dynamics with the aim of providing a proof of concept of how they may help obtaining empirical models of neuronal activity. We have generated the training data from mathematical models of neuronal activity. We have used both mechanistic (e.g. FitzHugh–Nagumo model) and simple biophysically meaningful models (e.g., Morris–Lecar or Wang model) exhibiting the most simple behaviors under constant applied current: silent states and spiking dynamics. We have followed this parsimonious approach in order to be able to describe the main obstacles that hinder the identification procedure.

We have used a specific type of artificial neural network, wavenets, consisting of a regression procedure on a fixed network architecture whose activation functions are wavelets; more precisely, they are based on the so-called Mallat multiresolution frames. The main advantage of using wavenets versus other types of ANNs is that the centers and supports of the network neurons (the frame) are already defined by the theory of wavelets. Hence, the unique parameters to be optimized are the coefficients of the approximation in its expression as a linear combination of wavelets. (Each node of the network is related to a specific wavelet.) We solve a least-squares optimization problem, which is linear in the parameters, thus ensuring a global solution. We believe that similar results can be also obtained with other types of ANNs; the challenge, in each case, is to refine the chosen method to obtain accurate results.

We have examined two different paradigms: the first one, which only aims to analyze the ANN’s ability to learn neuronal data, uses all model variables as input; the second one, on the other hand, considers only voltage data as input and is closely related to the ultimate goal of this research: to apply the methodology to experimental data. In Paradigm I, we have obtained an excellent performance. We have detected, however, a lower training capability when different timescales are present. This becomes evident when passing from two-dimensional to three-dimensional models by means of additional slow variables. We have resolved this problem by increasing the number of nodes (wavelons); of course, this scales up the computational demands, but allows to maintain the identification capability of the network. In order to train the wavenet, we have used data that spans up to 2500 s when the computational cost was low (basically, for 2D models). However, we think that there is room for a decrease of the training time, which would play in favor of the experimental conditions. The results for Paradigm II are not as accurate, but still satisfactory for the 2D models. In this case, we observe delays in the prediction that reduce performance, probably due to the fact that we take previous iterates of the voltage as input data at each time step. These inaccuracies could be mitigated by considering more iterates, although one has to be careful because this approach can lead to a sudden increase in computational cost. Before scaling up computational cost, it is worth exploring alternative options. We believe that, using techniques from control theory, it would be possible to build up a state observer to infer the non-measurable variables. This would allow to pass from Paradigm II to Paradigm I eventually improving the performance. However, pursuing this line goes beyond the scope of the current paper.

Even though we have restricted ourselves to models with simple dynamics, it is worth noting that the intervals of input currents used to train the network include both current levels for which the neuron is quiescent and current levels that elicit spikes and, moreover, as a result of applying non-constant input currents, we have also identified bursting-like or other input-induced complex behaviors. This implies frequent alternations between different topological attractors, thus showing the ability of our method to identify abrupt changes in the bifurcation diagram, from almost linear input–output relationships to highly nonlinear ones. Therefore, we think that the methodology presented is applicable to other neuron models or parameter regions encoding more sophisticated types of intrinsic dynamics (bistability, bursting, adaptation, mixed-mode oscillations, etc.); however, we must be cautious since the presence of multiple timescales will require improvements of the algorithms in order to increase the computational efficiency.

An open question that we would like to explore next (indeed, our ultimate goal) is what happens with real data. One would need to pharmacologically isolate a cell (or a population) and inject an input current (denoted as $I_{app}(t)$ throughout the paper), using, for example, a dynamic clamp technique, which should cover the range of eventual synaptic inputs. We would then obtain the voltage data, which could be used together with the signal $I_{app}(t)$ to train the wavenet. This is exactly what we have done in Paradigm II. In spirit, the results achieved in this paradigm are model-free except for the “nuance" that we have an a priori idea of the dimension of the system. Therefore, for real data, we would have to guess what the dimension of the underlying dynamics behind a membrane voltage or a firing rate time series is, or how many timescales they encode. It is difficult to answer these questions in general, but in specific cases one could try to increase the number of previous iterations used in Paradigm II, with the risk, of course, of excessively increasing the computational cost. We are optimistic about the potential success of this approach based on the results we are presenting.

Opening another focus of discussion, we are aware that the models obtained using the procedure presented in this paper are black boxes, blind to the biophysical features underlying the data. This circumstance constrains their use for further analysis and predictions since the general question behind this problem, “how does the wavenet change when modifying biophysical parameters?" is challenging and difficult. A preliminary sensitivity analysis in the FitzHugh–Nagumo model (not shown here) reveals a one-to-one relationship between some parameters of the model and the information encoded in the weights of the network (for instance the influence of the different levels of resolution used in the wavenet), but the results are not conclusive enough yet. Even though, we think that, because of its structure, wavenets may be more appropriate than other ANNs to tackle this challenge. Sensitivity analysis of deep neural networks (DNNs) has been recently studied in Shu and Zhu (2019), where the authors introduce a new perturbation manifold and its associated influence measure to quantify the sensitivity of DNNs to different perturbations. In fact, this problem connects with the concept of *explainability*, which is a current hot topic in machine learning, see Linardatos et al. (2020) for a recent review. We think that pursuing this direction would allow to provide hints of how to use biological constraints to construct the network; in other words, how to alter the trained neural network in order to mimic the variation of some biological meaningful parameters.

The above question poses also an interesting alternative: thanks to the analogy of neuronal dynamics with electrical circuits, which was Hodgkin and Huxley’s inspirational idea to come up with its famous model, one can assume that single neuron mathematical models have a particular shape, namely,

$$\begin{aligned} \left\{ \begin{array}{rl} C\,\dfrac{dv}{dt}=&{}-g_L(v-V_L)\\ &{}-\sum \limits _{\iota \in \Upsilon }\, g_{\iota }\,w_{\iota }^{p_{\iota }}(v-V_{\iota })+ I_{app},\\ &{}\\ \dfrac{dw_{\iota }}{dt}=&{}\dfrac{W_{\iota }(v)-w_{\iota }}{\tau _{\iota }(v)},\quad |\Upsilon | \approx \sharp \text{ ionic } \text{ channels, } \end{array} \right. \end{aligned}$$

where $|\Upsilon |$, $\{g_{\iota }\}_{\iota \in \Upsilon }$, $\{V_{\iota }\}_{\iota \in \Upsilon }$ and the parameters that define the functions $W_i(v)$ and $\tau _i(v)$ (typically, two and four, respectively, for each $\iota$) become the unknown parameters involved. For population models, as discussed in the Introduction, there are not comprehensive models as the Hodgkin–Huxley one, yet there are a limited number of approaches (firing rate, neural mass, neural fields, population density,...), which leads to a short list of candidate models. Then, an interesting alternative would be using machine learning methods (e.g., genetic algorithms) to identify parameters instead of doing a complete identification of the system, as we have performed in this work. Although this is not a new idea (see for instance (Schmidt and Lipson 2009) where the authors apply what they call *genetic programming*), the application to neural data would bring up “non-black" boxes which would allow a subsequent mathematical analysis using tools from dynamical systems. Besides, this would bring up a connection to the ambiguity of models: parameter searching algorithms could lead to different optima, that is, different parameter sets, with similar dynamics.

Another possible extension of our work is estimating the inputs from voltage (or other observable) data. Assuming that we have an accurate wavenet model, like the ones we have obtained in the present work, we can massively obtain voltage output data from prescribed current input traces (for instance, randomly generated). This procedure provides a way to build up a dataset of time-series pairs, formed by voltage and current traces; therefore, voltage traces can be thought as inputs and current traces as outputs. From this point on, it would be possible to identify the voltage–current relationship with a wavenet network that could be considered as the *inverse* of the original one. Of course, here the term “inverse" has to be taken carefully since it is not clear that two different current input traces could lead to the same voltage trace for the original model. In this possible extension, wavenet models take advantage with respect to classical differential equation models since they are more easily invertible. Preliminary tests have shown promising results, and it will be a line of continuation of the present work.

Recently, other works have appeared that use machine learning to understand neural dynamics. An excellent overview can be found in Saxe et al. (2021), where the authors highlight the incipient role of DNNs as promising theories of neural computations. They address interesting topics, some of them already mentioned above: Are biological signals related to activation of the nodes of the artificial network? Do the learning rule of the artificial networks explain learning rule at the biological level? We also emphasize the work of Beniaguev et al. (2020), where they introduce a novel approach to study neurons as sophisticated I/O information processing units by utilizing recent advances in the field of machine learning, by training DNNs to mimic the I/O behavior of a detailed nonlinear model of a layer 5 cortical pyramidal cell, receiving rich spatiotemporal patterns of input synapse activations. Another interesting application of machine learning tools to single-cell data is in spike detection (cell classification in order to sort neural action potentials) (Ekanadham et al. 2014).

Summing up, despite the computational challenges involved, our approach opens promising avenues when applied to real neurons, since it naturally leads to a heuristic model of a real neuron just stimulating it by means of protocols that allow to inject a prescribed input current trace (for instance, dynamic-clamp, a well-known electrophysiology protocol) and using this input current together with the output data to train the wavenet. In particular, this procedure will be able to *host* datasets into such heuristic models without needing to go through expensive and time-consuming biophysical experiments, as well as reusing existing data. This application extends to both single-cell data and population data obtained with modern recording methods and ultimately provides a strong predictive tool with extraordinary potential applications.

## Data Availability

The data supporting the findings of this study are available upon request from the authors.

## Neuron models

As benchmark neuron models, we consider several well-known models in computational neuroscience aiming at exploring diverse dynamical features, the most prominent being the presence of both quiescent states and regular spiking, and timescale separation. They have been integrated using the Euler method in order to maintain the computational algorithm simple enough and to obtain reliable predictions from our ANN. In conductance-based models, described in Sects. A.1 and A.4, the time step has been taken to be $\Delta t=0.05$ ms and $\Delta t=0.005$ ms, respectively, both close to usual experimental sampling frequencies. For the FitzHugh–Nagumo models, described in Sects. A.2 and A.3, a dimensionless time step of $\Delta t = 0.05$ has been taken, having ensured that using shorter time steps provides similar qualitative results.

Note that, despite the poor accuracy of the Euler method compared to higher-order ODE integrators, it is not an essential issue here because it is only used to generate the input data and the purpose of the paper is to identify the simulated data with a neural network. Therefore, it is not relevant how the target data are created. In other words, one can think that our data source is not the continuous model under consideration but the “discrete model obtained by integrating the continuous model with the Euler method".

### Morris–Lecar

The Morris–Lecar model was proposed in Morris and Lecar (1981). While it models a fundamental type of neural dynamics, it is still feasible to make a qualitative analysis of it, see for instance (Rinzel and Ermentrout 1998). The dynamics of the neuron are modeled by a continuous-time dynamical system composed of the current-balance equation for the membrane potential, $v = v(t)$, and the $K^+$ gating variable $0\le w = w(t) \le 1$, which represents the probability of the $K^+$ ionic channel to be active:

A.1 $$\begin{aligned} \begin{array}{rl} C_m\displaystyle \frac{dv}{dt}&{}=-I_L-I_K-I_{Ca}+I_{app}, \\ {} &{} \\ \displaystyle \frac{dw}{dt} &{}= \phi \dfrac{w_\infty (v)-w}{\tau _w(v)}, \end{array} \end{aligned}$$

where the leakage, calcium, and potassium currents are defined as $I_L=g_L\,(v-E_L)$, $I_{Ca}=g_{Ca}\,m_{\infty }(v)\,(v-E_{Ca})$, and $I_K = g_K \,w\,(v-E_K)$, respectively; $g_L$, $g_{Ca}$ and $g_K$ are the maximal conductances of each current, whereas $E_L$, $E_{Ca}$ and $E_K$ denote the Nernst equilibrium potentials, for which the corresponding current is zero, also known as *reversal potentials*. The constant $C_m$ is the membrane capacitance, $\phi$ is a dimensionless constant and $I_{app}$ represents the (externally) applied current. The auxiliary functions of the model are given by:

A.2 $$\begin{aligned} \begin{array}{cc} m_{\infty }(v) &{}= \left( 1+ \tanh ((v-V_1)/V_2)\right) /2,\\ w_{\infty }(v) &{}= \left( 1+ \tanh ((v-V_3)/V_4)\right) /2,\\ \tau _w(v) &{}= \left( \cosh ((v-V_3)/(2\,V_4))\right) ^{-1}. \end{array} \end{aligned}$$

In our computations, we have used the following set of parameters (units are expressed for each parameter type), see Rinzel and Ermentrout (1998):

A.3 $$\begin{aligned} \begin{array}{l} C_m=20\,(\upmu \text{ F/cm}^2), \phi =1/15,\\ E_L=-60,\, E_K=-84,\\ E_{Ca}=120\ (\text {mV}),\\ V_1=-1.2,\, V_2=18,\\ V_3=12,\, V_4=17.4\ (\text {mV}),\\ g_L=2,\, g_K=8.0,\, g_{Ca}=4.0\ (\text {mS/cm}^2). \end{array} \end{aligned}$$

Figure 20 shows the bifurcation diagram of system (A.1) in terms of the parameter $I_{app}$. In the experiments, a prescribed $I_{app}(t)$ that spans from 20 to 80 $\mu A/\text{cm}^2$ was used. Note that for $I_{app}$ below $I_{bif}\approx 39.9632$, there is one attractor, which is an equilibrium point of the system, while for $I_{app}\in (I_{bif},80)$, also there is a unique attractor, which is a limit cycle (only the maximal value of the variable *v* on the limit cycle is shown).

### FitzHugh–Nagumo model

The FitzHugh–Nagumo model, derived independently by FitzHugh (1961) and Nagumo et al. (1962), is a reduced version of the Hodgkin–Huxley model (Hodgkin and Huxley 1952) that captures its key features but is analytically more tractable. By means of steady-state assumptions and approximating invariant manifolds, the four variables of the Hodgkin–Huxley model are reduced to two, essentially a fast variable related to the membrane potential and a slow variable related to the channel dynamics. Namely,

A.4 $$\begin{aligned} \begin{array}{rl} \displaystyle \frac{dv}{dt}&{}= -f(v) + w + I_{app}, \\ {} &{} \\ \displaystyle \frac{dw}{dt}&{}= \epsilon \,(-v-\gamma \,w), \end{array} \end{aligned}$$

where $f(v) = v\,(v-1)\,(v-a)$. Unless otherwise stated, we will take the parameter values $a=0.14$, $\gamma =2.54$ and $\epsilon =0.1$. In Fig. 21, we show the bifurcation diagram of system (A.4) in terms of $I_{app}$ for this choice of parameters.

In fact, the system is a caricature of the original Hodgkin–Huxley model aiming at capturing the geometry and relative position of the nullclines, as well as the timescales.

### FitzHugh–Nagumo–Rinzel model

In order to test the effect of adding a third variable with a slowest time scale, we consider an extension of the Fitzhugh–Nagumo model proposed by Rinzel in 1987, see Rinzel (1987) and Izhikevich (2001), departing from (A.4) and adding an additional slow variable. We consider the system

A.5 $$\begin{aligned} \begin{array}{rl} \displaystyle \frac{dv}{dt}&{}= -f(v) + w -\alpha \, y+ I_{app}, \\ &{} \\ \displaystyle \frac{dw}{dt}&{}= \epsilon \,(-v-\gamma \,w),\\ &{} \\ \displaystyle \frac{dy}{dt}&{}= \delta \,(c-v-d\,y), \end{array} \end{aligned}$$

where $f(v) = v\,(v-1)\,(v-a)$ as in (A.4). Unless otherwise stated, we will take the parameter values $a=0.14$, $\gamma =2.54$, $\epsilon =0.1$, $\delta =0.01$, $c=-0.775$ and $d=1$. In Fig. 22, we show the bifurcation diagram of system (A.5) in terms of $I_{app}$ and $\alpha$ for this choice of parameters; panels (c) and (d) are biparametric bifurcation diagrams intended to include the parameter $\alpha$.

Our choice of parameters avoids bursting because we are more interested in exploring the difficulties that our neural network encounters when trying to identify three-dimensional systems with an extra timescale. The extended FitzHugh–Nagumo system we consider is graded by the parameter $\alpha$ which modulates the effect of the slow variable *y*: for $\alpha =0$ we have a decoupled system but already a slow dynamics, while for $\alpha >0$ and small enough, the model still keeps the essence of the FitzHugh–Nagumo system (A.4), that is, the presence of an attracting limit cycle in some region of the $I_{app}$ bifurcation diagram.

### Wang model of a pyramidal neuron

In order to test more realistic models, we use a conductance-based model that describes the activity of a cortical pyramidal cell. We adapt a simplification of Traub’s model, borrowing the values for the characteristic conductances from Wang (1998). In order to avoid complicated dynamics in this testing stage of our methods, we only consider the soma compartment from Wang’s model and neglect the dendritic one; moreover, we have removed the differential equation for the calcium concentration and brought the gating variable *m* to its steady-state, $m=m_{\infty }(v)$. The resulting model contains both a sodium and a potassium current, which drive the membrane potential during spiking.

A.6 $$\begin{aligned} \displaystyle C_m \frac{dv}{dt}=-I_L-I_{Na}-I_K+I_{app}. \end{aligned}$$

As in the other models, equation (A.6) contains a constant applied current ($I_{app}$). The ionic currents in (A.6) are given by:

$$\begin{aligned} \left\{ \begin{array}{rl} I_L&{}=g_L\ (v-V_L),\\ &{}\\ I_{Na}&{}=g_{Na}\ m_{\infty }^3(v)\ h\ (v-V_{Na}),\\ &{}\\ I_K&{}=g_K\ n^4\ (v-V_K).\\ \end{array} \right. \end{aligned}$$

The gating variables *h* and *n* satisfy the usual type of differential equation:

A.7 $$\begin{aligned} \begin{array}{rl} \displaystyle \displaystyle \frac{dw}{dt}&{}=\phi \ [\alpha _w(v)\ (1-w)-\beta _w(v)\ w], \\ \end{array} \end{aligned}$$

where *w* represents either *h* or *n*, and

A.8 $$\begin{aligned} \begin{array}{rl} \alpha _h(v)&{}=0.07\ \exp {(-(v+50)/10)},\\ \beta _h(v)&{}=\displaystyle \frac{1}{1+\exp {(-0.1\ (v+20))}},\\ \alpha _n(v)&{}=-0.01 \displaystyle \frac{v+34}{\exp {(-0.1\ (v+34))}-1},\\ \beta _n(v)&{}=0.125\ \exp {(-(v+44)/25)},\\ \alpha _m(v)&{}=-0.1\displaystyle \frac{v+33}{\exp {(-0.1\ (v+33))}-1},\\ \beta _m(v)&{}=4\ \exp {(-(v+58)/12)},\\ m_{\infty }(v)&{}= \alpha _m(v)/(\alpha _m(v)+\beta _m(v)). \end{array} \end{aligned}$$

In our computations, we have used the following set of parameter values (units are expressed for each parameter type):

A.9 $$\begin{aligned} \begin{array}{l} C_m=1\ (\mu F/cm^2), \phi =4, \\ V_L=-65, V_{Na}=55, V_K=-80,\\ g_L=0.1,\ g_{Na}=45,\\ g_K=18\ (\text {mS/cm}^2).\\ \end{array} \end{aligned}$$

Figure 23 shows the bifurcation diagram in terms of parameter $I_{app}$.

## Declarations

### Code availability

The developed code is available on GitHub: https://github.com/pau-3i8/Wavenet_research
